# Mitochondrial genomes of four slug moths (Lepidoptera, Limacodidae): Genome description and phylogenetic implications

**DOI:** 10.1002/ece3.11319

**Published:** 2024-04-29

**Authors:** Feng Jiang, Xu‐Dong Yu, En‐Tao Sun, Sheng‐Li Gu, Ying Liu, Ting Liu

**Affiliations:** ^1^ School of Basic Medical Sciences Wannan Medical College Wuhu China; ^2^ Anhui Provincial Key Laboratory of Biological Macro‐Molecules Wuhu China; ^3^ School of Laboratory Medicine Wannan Medical College Wuhu China; ^4^ School of Medical Information Wannan Medical College Wuhu China

**Keywords:** phylogenetic relationship, start codon, structural characteristics, systematics, Zygaenoidea

## Abstract

The family Limacodidae belongs to the superfamily Zygaenoidea, which includes 1672 species commonly referred to as slug moths. Limacodidae larvae are major pests for many economically important plant species and can cause human dermatitis. At present, the structure of the mitochondrial genome (mitogenome), phylogenetic position, and adaptive evolution of slug moths are poorly understood. Herein, the mitogenomes of *Parasa lepida*, *Phlossa conjuncta*, *Thosea sinensis*, and *Setora sinensis* were sequenced and compared with other available mitogenome sequences to better characterize the mitogenomic diversity and evolution of this moth family. The mitogenomes of *P. lepida*, *P. conjuncta*, *T. sinensis*, and *S. sinensis* were confirmed to be circular in structure with lengths of 15,575 bp, 15,553 bp, 15,535 bp, and 15,529 bp, respectively. The Limacodidae mitogenomes exhibited similar nucleotide composition, codon usage, RNA structure, and control region patterns, indicating the conservation of the mitogenome in the family Limacodidae. A sliding window, Ka/Ks, and genetic distance analyses revealed that the *atp8* and *nad6* genes exhibited the highest levels of variability and the most rapid evolutionary rates among the 13 protein‐coding genes (PCGs) encoded in these Limacodidae mitogenomes, suggesting that they may offer value as candidate DNA markers. The phylogenetic analysis recovered the overall relationship as Tortricoidea + (Sesiidae + (Zygaenoidea + (Cossoidea/+Choreutoidea + (others)))). Within Zygaenoidea, Limacodidae was recovered as monophyletic, and the phylogenetic relationships were recovered as (Phaudidae + Zyganidae) + Limacodidae in all six phylogenetic trees. The analysis indicated that *P. lepida*, *P. conjuncta*, *T. sinensis*, and *S. sinensis* are members of the Limacodidae.

## Graphical abstract

In this study, we sequenced the mitochondrial genomes of *Parasa lepida*, *Phlossa conjuncta*, *Thosea sinensis*, and *Setora sinensis*, and performed the first comparative genomic analyses of Limacodidae. The use of the ATT start codon by the *cox1* gene in *P. lepida* is the first documented use of this start codon in Limacodidae mitogenomes. Within Zygaenoidea, Limacodidae was recovered as monophyletic, and the phylogenetic relationships were recovered as (Phaudidae + Zyganidae) + Limacodidae in all six phylogenetic trees.

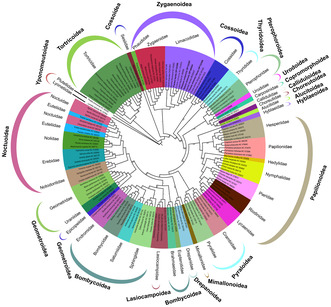

## 1 INTRODUCTION

Given their status as eukaryotic organelles that originally evolved from endogenous symbiotic bacteria, mitochondria have been major targets of research interest for over a century (Andersson et al., 1998; Jacobs, 2023; Sagan, 1967). The mitochondrial genome (mitogenome) in insects consists of a closed double‐stranded circular DNA loop that offers substantial value to studies of genetic evolution, species origins, population biology, phylogenetic relationships, and taxonomic classification owing to the high mutation rates, rapid evolution, and low recombination rates to which mitogenomic DNA is subjected (Chen et al., 2022; Sankoff et al., 1992; Tao et al., 2023; Wolstenholme, 1992).

Over 160,000 lepidopteran species have been described to date, making it the second‐largest insect order after Coleoptera (Kawahara et al., 2019; Li et al., 2021; Mullen & Zaspel, 2019; Zheng et al., 2018). The superfamily Zygaenoidea consists of approximately 3300 species with no clearly defined unique characteristics, which is also the case for other families within the suborder Ditrysia (Heikkila et al., 2015; Mitter et al., 2017; van Nieukerken et al., 2011). For this reason, no uniform classification standards have been used in previous studies, with different researchers having used varying criteria to divide the superfamily Zygaenoidea into 7–13 families (Niehuis et al., 2006; Scoble, 1992; van Nieukerken et al., 2011; Zhang, Li, et al., 2020). Alberti (1954) first divided the zygaenid moths into seven subfamilies based on morphological characteristics, namely Anomoeotinae, Chalcosiinae, Charideinae, Himantopterinae, Phaudinae, Procridinae, and Zygaeninae. Subsequently, Fletcher and Nye (1982) removed the subfamilies Himantopterinae and Anomoeotinae from the Zygaenidae and raised them to family‐level classification status. Minet (1991) further classified Charideinae into the family Thyrididae. In addition, Naumann et al. (1991) reclassified the zygaenid moths into 12 families including Epipyropidae, Cyclotornidae, Dalceridae, and Heterogynidae. Recently, Niehuis et al. (2006) constructed a phylogenetic relationship of the superfamily Zygaenoidea based on the ND1, tRNA‐Leu, 16S rRNA, tRNA‐Val, and 12S rRNA partial mitochondrial gene fragments and 18S and 28S rRNA two nuclear gene fragments, and the superfamily Zygaenoidea was divided into seven families: Heterogynidae, Himantopteridae, Lacturidae, Limacodidae, Phaudidae, Somabrachyidae, and Zyganidae. Simultaneously, the family Zyganidae underwent a division into four subfamilies: Callizygaeninae, Chalcosiinae, Procridinae, and Zygaeninae four subfamilies.

The slug moth family Limacodidae (1672 species) belongs to the superfamily Zygaenoidea (van Nieukerken et al., 2011). They are a class of plant pests in subtropical and tropical regions. The taxonomic status of Limacodidae within Lepidoptera has been controversial. Previously, Limacodidae was classified under Tineoidea or Psychoidea. However, Brock (1971) classified it as a member of the superfamily Cossoidea based on the morphological characteristics of the adult thorax and forewings. Other researchers further confirmed and supported this classification (Fletcher & Nye, 1982; Heppner, 1998; Holloway, 1986). However, Common (1975) placed it in Zygaenoidea based on immature‐stage characters. Epstein (1996) classified Limacodidae, Aididae, Dalceridae, Megalopygidae, and Somabrachyidae as monophyletic groups based on the characteristics of adults and larvae, and classified them into Zygaenoidea. Thus, the phylogenetic relationship of Zygaenoidea and its internal phylogeny remains unclear.

**FIGURE 1:**
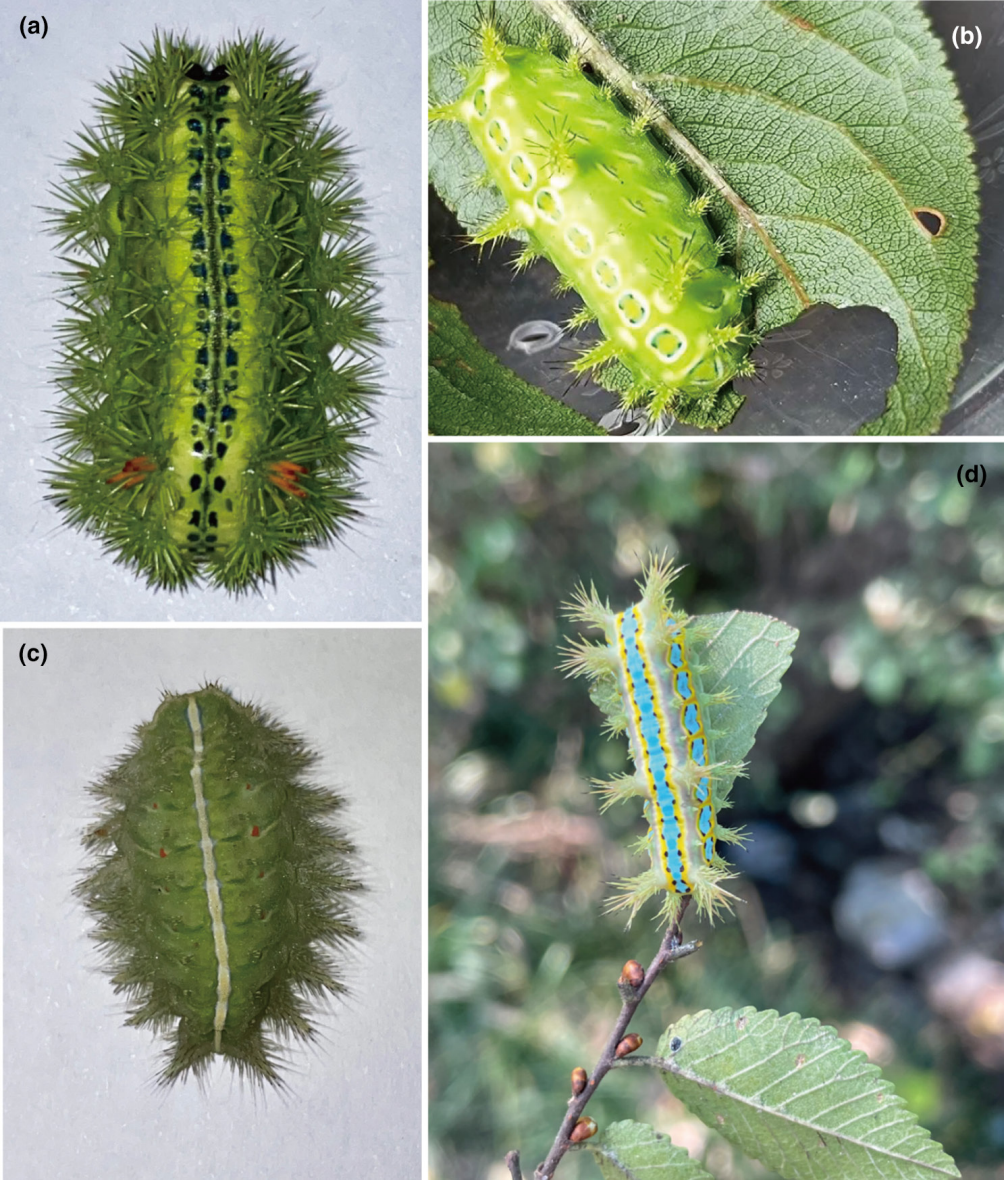
Larvae of *Parasa lepida* (a), *Phlossa conjuncta* (b), *Thosea sinensis* (c), and *Setora sinensis* (d) collected by Yu XD. Photograph by Yu XD.

As of January 2023, more than 1000 Lepidoptera mitogenomes have been published. However, only 17 are available for species in the superfamily Zygaenoidea. Due to limitations of molecular data, phylogenetic studies of the superfamily Zygaenoidea have long remained at the morphological level. To increase the existing genomic information for these insects, the present work was conducted to sequence and annotate the mitogenomes of four slug moths belonging to the family Limacodidae (Figure 1). The mitogenomes of *Phlossa conjuncta* and *Setora sinensis* sequenced herein are the first time for the genera *Phlossa* and *Setora*. In contrast, *Parasa lepida* is the second species sequenced in the genus *Parasa*, and an additional resequencing of *Thosea sinensis* was performed for these analyses (Bian et al., 2020). The analysis and comparison of the mitogenomes of different slug moth species belonging to the family Limacodidae established a foundation for research focused on the genetic structure of this family. *T. sinensis* is one of the dominant species among the plant‐damaging slug moth species (Xiao et al., 2020). Therefore, resequencing the *T. sinensis* mitogenome will provide a valuable reference for future research on the genetic diversity, population structure, and origin of this species. To gain a deeper understanding of the superfamily Zygaenoidea and its internal phylogenetic structure, 144 Apoditrysia mitogenomes from three datasets were utilized to reconstruct six phylogenetic trees. Two species from the superfamily Yponomeutoidea (*Lyonetia clerkella* and *Plutella armoraciae*) were used as outgroups, which served as a basis for future research on the phylogenetic relationships among members of the superfamily Zygaenoidea.

## 2 MATERIALS AND METHODS

### 2.1 Sample collection and DNA extraction

The larvae samples of *P. lepida*, *P. conjuncta*, *T. sinensis*, and *S. sinensis* were collected from different provinces in China (Zhejiang, Henan, and Anhui) in 2021 (Table 1). A thorax muscle tissue sample from each fresh specimen was stored in 100% ethanol at −20°C for future use. Morphological characteristics were used to identify these specimens (Epstein, 1996; Wang & Wang, 2009), and voucher specimens were kept in the Department of Medical Parasitology, Wannan Medical College, Anhui Province, China. Based on the provided protocols, the DNeasy Blood & Tissue Kit (Qiagen, Hilden, Germany) was used to extract total DNA from these samples. The extracted DNA was stored at −20°C.

**TABLE 1:** Sampling details for four specimens used in this study.

Species	Location	Collection date
*P. lepida*	Hangzhou City, Zhejiang Province (30°37′ N, 120°04′ E)	October 5, 2021
*P. conjuncta*	Nanyang City, Henan Province (32°99′ N, 111°28′ E)	October 14, 2021
*T. sinensis*	Nanyang City, Henan Province (32°99′ N, 111°28′ E)	October 14, 2021
*S. sinensis*	Wuhu City, Anhui Provinces (31°28′ N, 118°36′ E)	September 5, 2021

### 2.2 Sequencing and assembly

Genepioneer Biotechnologies Co. Ltd (Nanjing, China) prepared all DNA libraries and used an Illumina NovaSeq instrument for the 150 bp paired‐end sequencing of these libraries. Fastp (Chen et al., 2018) (https://github.com/OpenGene/fastp) was used to identify sequencing adapter and primer sequences and to filter out any raw reads with an N‐base content >5 or an average quality <Q5. In total, 17,669,659 (*P. lepida*), 18,778,151 (*P. conjuncta*), 20,825,481 (*T. sinensis*), and 19,736,209 (*S. sinensis*) clean read pairs were retained after quality control filtering, with clean rates of 99.75%, 99.18%, 99.21%, and 99.66%, respectively. These reads were aligned with the local database using Bowtie2 v2.2.4 (Langmead & Salzberg, 2012) (http://bowtie‐bio.sourceforge.net/bowtie2/index.shtml) to obtain seed sequences. The seed sequences for these four mitogenomes were assembled with SPAdes v3.10.1 (Bankevich et al., 2012), and contigs were obtained with a k‐mer iterative extend seed. SSPACE v2.0 (Boetzer et al., 2011) (https://www.baseclear.com/services/bioinformatics/basetools/sspace‐standard/) was then used to connect these contig sequences to establish scaffolds, and gaps in the scaffold sequences were subsequently filled with Gapfiller v2.1.1 (Boetzer & Pirovano, 2012) (https://sourceforge.net/projects/gapfiller/). Subsequent quality control assessments were conducted on the assembled mitogenomes using the related species *Neptis philyra* (GenBank accession number: MW_813978.1).

### 2.3 Mitogenomic annotation and analysis

The annotation of assembled mitogenomic sequences was performed with the MITOS tool (Bernt et al., 2013) (Parameters: E‐value Exponent = 5, Maximum Overlap = 100, ncRNA overlap = 100) (http://mitos2.bioinf.uni‐leipzig.de), and the annotation results were manually corrected with Geneious v8.0.4 (Kearse et al., 2012) based on the related species *Neptis philyra*, *Parasa consocia* (GenBank accession numbers: NC_034993.1), and *Narosa nigrisigna* (GenBank accession numbers: NC_041304.1), respectively. Furthermore, the tRNAs were verified using the tRNAscan‐SE tool (Lowe & Chan, 2016) (http://lowelab.ucsc.edu/tRNAscan‐SE/). The final annotated mitogenomes were mapped with OGDRAW (Greiner et al., 2019) (https://chlorobox.mpimp‐golm.mpg.de/OGDraw.html). In addition, a reannotation of nine mitogenomes was performed for phylogenetic and comparative genomic analyses. Their annotated information is detailed in Table S1. Base composition analyses were conducted using Phylosuite v1.2.3 (Zhang, Gao, et al., 2020) for the entire mitogenome, 13 PCGs, and two rRNAs. The base composition of concatenated PCGs concatenated 22 tRNAs, and three codon positions were assessed with DNAStar. Repeat sequences in the CR were identified with Tandem Repeats Finder (Benson, 1999) (http://tandem.bu.edu/trf/trf.html), while AT‐ and GC‐skewness were calculated as follows: AT‐skew = (A – T)/(A + T) and GC‐skew = (G – C)/(G + C) (Perna & Kocher, 1995). The software DnaSP v6 (Rozas et al., 2017) was used to determine nucleotide diversity (π), as well as the nonsynonymous (Ka) and synonymous (Ks) substitution rates for 13 PCGs. Average genetic distance values for the 13 PCGs were calculated with MEGA X (Kumar et al., 2018) using a Kimura 2‐parameter model for 11 available Limacodidae mitogenomes. The *P. lepida*, *P. conjuncta*, *T. sinensis*, and *S. sinensis* mitogenomic sequences were submitted to GenBank with the accession numbers OP132386 – OP132388 and OP160524, respectively.

### 2.4 Primer design, PCR amplification, and sequencing

To validate the start codon of the *cox1* gene of *P. lepida*, we designed two pairs of primers using Primer Premier v5.0 (Premier Biosoft, Palo Alto, CA, USA): 1L_F: 5′‐TCGCTTAATAACTCAGCCATTT‐3′, 1L_R: 5′‐GCTATGTCTGGAGCACCAAGTA‐3′; 2L_F: 5′‐CTCTACTTTCTATTTTACTCCTTTT‐3′, 2L_R: 5′‐ATCCTGGATTACCTAATTCAGC‐3′. A total of 25 μL of the PCR reaction system was used (12.5 μL of Prime STAR Max Premix (2×) (TaKaRa, Beijing, China), 2 μL of template DNA, 1 μL each of 10 μM forward and reverse primers, and the rest was made up with double distilled water). PCR amplification was performed under the following conditions: 95°C for 5 min, 35 cycles of 95°C for 30 s, 50–52°C for 60 s, and 72°C for 60 s. PCR products were detected by 1% agarose gel electrophoresis and sent to General Biosystems (Anhui) Co., Ltd for Sanger sequencing. Then, the sequencing results were compared to *P. lepida* mitogenome using DNAMAN version 6.0 (Lynnon Biosoft, USA).

### 2.5 Phylogenetic analyses

Phylogenetic trees for 144 Apoditrysia mitogenomes were reconstructed to confirm the superfamily Zygaenoidea and its internal phylogenetic status using Bayesian Inference (BI) and maximum likelihood (ML) approaches based on the following datasets: (I) PCGs_rRNA dataset (13 PCGs and two rRNA genes), (II) PCGs_rRNA_codPosOneTwo (first and second codon positions of 13 PCGs and two rRNA genes), and (III) PCGs_AA dataset (amino acid sequences of 13 PCGs). The outgroups used for these phylogenetic analyses were *L. clerkella* (NC_037944.1) and *P. armoraciae* (NC_064059.1) from the superfamily Yponomeutoidea (Table S2). The PCG and RNA sequences from these mitogenomes were extracted with Phylosuite v1.2.3 (Zhang, Gao, et al., 2020). The nucleotide and amino acid sequences for the 13 PCGs and the nucleotide sequences for the RNAs were aligned with MAFFT v7.313 (Katoh & Standley, 2013) (Code table: 5 Invertebrate mitochondrial). Nucleotide sequence alignment results for these 13 PCGs were optimized with MACSE v2.03 (Ranwez et al., 2018), and ambiguously aligned fragments from these 13 alignments were removed in batches with Gblocks 0.91b (Talavera & Castresana, 2007) in Phylosuite v1.2.3. Any gaps in RNA and AA sequences were removed with TrimAl v1.2rev57 (Capella‐Gutierrez et al., 2009), and the best‐fit partition model was selected using a greedy algorithm and the Akaike information criterion (AIC) for BI analyses in PartitionFinder2 v2.1.1 (Lanfear et al., 2017). BI trees were calculated inferred with MrBayes 3.2.6 (Ronquist et al., 2012), with 10,000,000 generations and a partition model using two parallel runs and four independent Markov chains, performing sampling every 1000 generations. After reaching an average standard deviation for split frequencies <0.01, the first 25% of the sampled data were discarded, with the remaining trees then being used to represent posterior probability (PP) values. The best‐fit partition model (Edge‐linked) was selected using ModelFinder v2.2.0 (Kalyaanamoorthy et al., 2017) by a distribution‐free rate model using the Bayesian information criterion for ML analyses. ML trees were calculated with IQ‐TREE v1.6.8 (Nguyen et al., 2015). Using an edge‐linked partition model, 5000 ultrafast bootstrap replicates were used to assess bootstrap support (BS) (Minh et al., 2013). The best‐fit partition model details for each tree are provided in Table S3. ITOL (Letunic & Bork, 2021) was used to draw the final phylogenetic trees.

## 3 RESULTS

### 3.1 Mitogenomic organization and base composition

**FIGURE 2:**
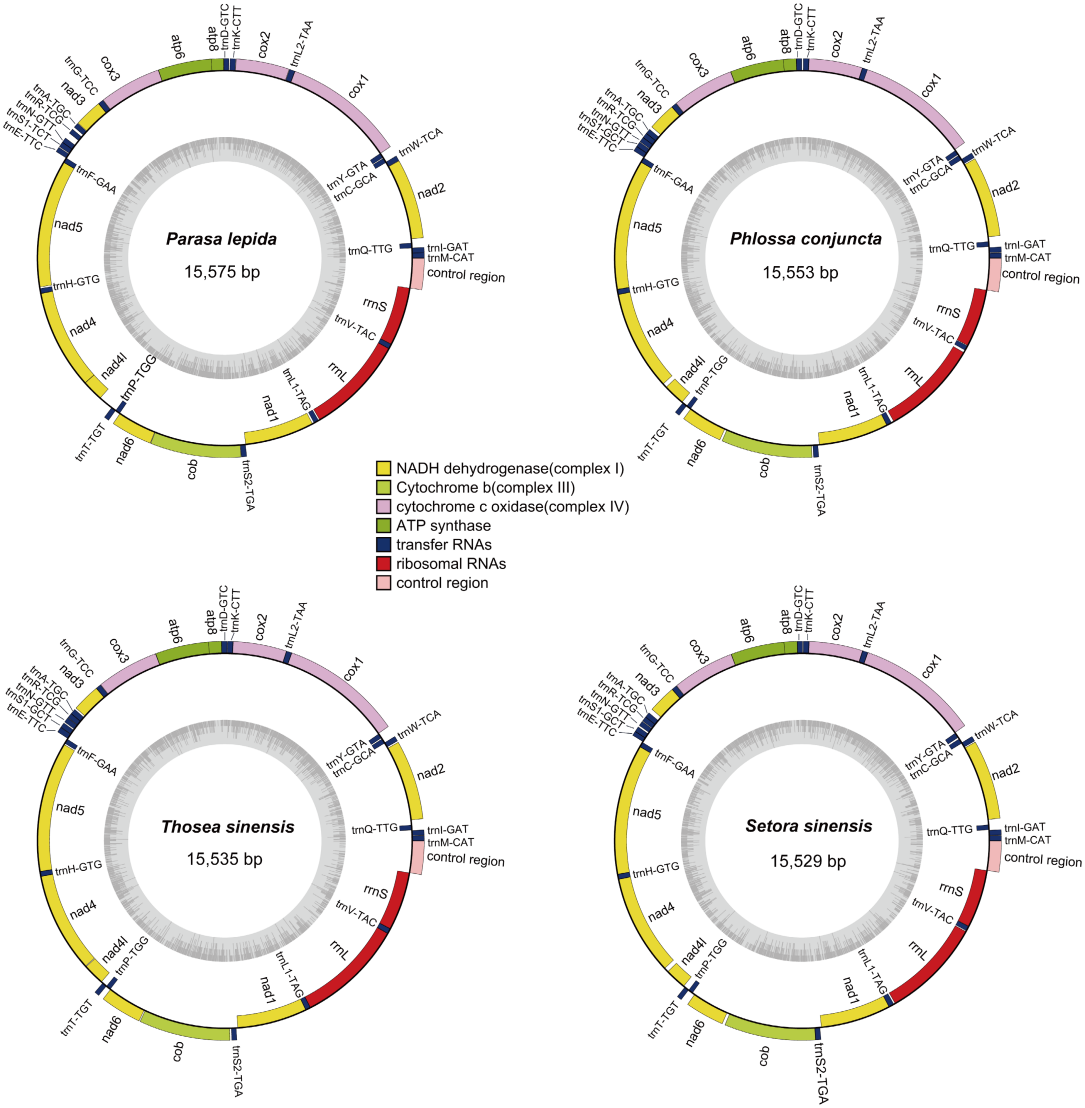
Gene maps for the *Parasa lepida*, *Phlossa conjuncta*, *Thosea sinensis*, and *Setora sinensis* mitogenomes. Genes inside and outside the circle are respectively located on the N‐strand and the J‐strand, respectively.

After sequencing was completed, the mitogenomes of *P. lepida*, *P. conjuncta*, *T. sinensis*, and *S. sinensis* were confirmed to be circular in structure with respective lengths of 15,575 bp, 15,553 bp, 15,535 bp, and 15,529 bp (Figure 2). These results were largely consistent with those for other published Limacodidae mitogenomes, which are 15.2–15.7 kb (Table S4). These four mitogenomes encode 37 total genes, including 22 tRNAs, 13 PCGs, and two rRNAs (*rrnL* and *rrnS*). Of these, 14 tRNAs and 9 PCGs were found to be encoded on the majority strand (J‐strand), while the remaining genes were encoded on the minority strand (N‐strand) (Table 2). Each of these four mitogenomes was also found to include a noncoding A + T‐control region (CR) located in the *rrnS* and the *trnM* genes from 393 to 425 bp in length.

**TABLE 2:** The mitogenomic organization of *Parasa lepida*, *Phlossa conjuncta*, *Thosea sinensis*, and *Setora sinensis* (in order).

Gene	Position		Size (bp)	Intergenic nucleotides	Codon		Strand
	From	To			Start	Stop	
*trnM*	1/1/1/1	68/66/66/68	68/66/66/68	0/0/0/0			J
*trnI*	66/73/69/71	135/141/137/140	70/69/69/70	−3/6/2/2			J
*trnQ*	133/151/142/145	201/219/210/213	69/69/69/69	−3/9/4/4			N
*nad2*	253/279/274/280	1263/1292/1278/1281	1011/1014/1005/1002	51/59/63/66	ATT/ATT/ATT/ATT	TAA/TAA/TAA/TAA	J
*trnW*	1266/1306/1295/1294	1333/1373/1362/1361	68/68/68/68	2/13/16/12			J
*trnC*	1326/1366/1355/1354	1396/1436/1422/1422	68/71/68/69	−8/−8/−8/−8			N
*trnY*	1403/1450/1436/1444	1469/1515/1502/1515	67/66/67/72	9/13/13/21			N
*cox1*	1467/1527/1507/1520	3003/3062/3037/3050	1537/1536/1531/1531	−3/11/4/4	ATT/CGA/CGA/CGA	T/TAA/T/T	J
*trnL2*	3004/3058/3038/3051	3075/3125/3105/3118	72/68/68/68	0/−5/0/0			J
*cox2*	3079/3126/3106/3119	3754/3801/3781/3794	676/676/676/676	3/0/0/0	ATA/ATA/ATA/ATA	T/T/T/T	J
*trnK*	3758/3802/3782/3795	3829/3872/3852/3866	72/71/71/72	3/0/0/0			J
*trnD*	3841/3890/3856/3874	3907/3958/3924/3940	67/69/69/67	11/17/3/7			J
*atp8*	3908/3959/3925/3941	4066/4123/4089/4108	159/165/165/168	0/0/0/0	ATT/ATT/ATT/ATT	TAA/TAA/TAA/TAA	J
*atp6*	4060/4117/4083/4102	4737/4794/4760/4779	678/678/678/678	−7/−7/−7/−7	ATG/ATG/ATG/ATG	TAA/TAA/TAA/TAA	J
*cox3*	4737/4794/4761/4779	5522/5579/5546/5564	786/786/786/786	−1/−1/0/−1	ATG/ATG/ATG/ATG	TAA/TAA/TAA/TAA	J
*trnG*	5525/5582/5549/5567	5592/5647/5614/5633	68/66/66/67	2/2/2/2			J
*nad3*	5593/5648/5615/5634	5946/6001/5968/5987	354/354/354/354	0/0/0/0	ATT/ATT/ATT/ATT	TAA/TAA/TAA/TAA	J
*trnA*	5951/6039/5989/6042	6015/6104/6058/6109	65/66/70/68	4/37/20/54			J
*trnR*	6046/6105/6059/6109	6111/6169/6122/6172	66/65/64/64	30/0/0/−1			J
*trnN*	6186/6170/6125/6178	6252/6235/6190/6242	67/66/66/65	74/0/2/5			J
*trnS1*	6253/6241/6205/6262	6319/6306/6270/6329	67/66/66/68	0/5/14/19			J
*trnE*	6328/6307/6272/6330	6393/6372/6341/6388	66/66/70/59	8/0/1/0			J
*trnF*	6392/6378/6350/6397	6458/6445/6416/6464	67/68/67/68	−2/5/8/5			N
*nad5*	6459/6446/6432/6465	8172/8192/8174/8208	1714/1747/1743/1744	0/0/15/0	ATT/ATT/ATT/ATA	T/T/TAA/T	N
*trnH*	8191/8193/8175/8209	8258/8260/8240/8275	68/68/66/67	18/0/0/0			N
*nad4*	8259/8261/8241/8276	9594/9599/9579/9614	1336/1339/1339/1339	0/0/0/0	ATG/ATG/ATG/ATG	T/T/T/T	N
*nad4L*	9594/9656/9587/9672	9878/9946/9874/9959	285/291/288/288	−1/56/7/57	ATA/ATG/ATG/ATG	TAA/TAA/TAA/TAA	N
*trnT*	10,074/9960/9896/9977	10,139/10,023/9959/10,040	66/64/64/64	195/13/21/17			J
*trnP*	10,140/10,024/9960/10,041	10,205/10,088/10,025/10,104	66/65/66/64	0/0/0/0			N
*nad6*	10,208/10,091/10,028/10,137	10,732/10,615/10,552/10,631	525/525/525/495	2/2/2/32	ATT/ATA/ATT/ATT	TAA/TAA/TAA/TAA	J
*cytb*	10,736/10,643/10,565/10,666	11,884/11,791/11,716/11,816	1149/1149/1152/1151	3/27/12/34	ATG/ATG/ATG/ATG	TAA/TAA/TAA/TA	J
*trnS2*	11,887/11,808/11,736/11,817	11,955/11,875/11,802/11,884	69/68/67/68	2/16/19/0			J
*nad1*	11,956/11,894/11,821/11,903	12,889/12,829/12,756/12,841	934/936/936/939	0/18/18/18	ATT/ATG/ATG/ATG	T/TAA/TAA/TAA	N
*trnL1*	12,912/12,831/12,757/12,843	12,980/12,899/12,823/12,914	69/69/67/72	22/1/0/1			N
*rrnL*	12,996/12,932/12,824/12,933	14,325/14,247/14,257/14,281	1330/1316/1434/1349	15/32/0/18			N
*trnV*	14,326/14,280/14,258/14,292	14,390/14,345/14,327/14,357	65/66/70/66	0/32/0/10			N
*rrnS*	14,392/14,351/14,328/14,359	15,176/15,128/15,111/15,136	785/778/784/778	1/5/0/1			N
*CR*	15,177/15,129/15,112/15,137	15,575/15,553/15,535/15,529	399/425/424/393	0/0/0/0			

Abbreviations: J, majority strand (J‐strand); N, minority strand (N‐strand).

The base composition was established for each of these four mitogenomes, including *P. lepida* (T = 40.5%, C = 12.6%, A = 39.6%, G = 7.4%), *P. conjuncta* (T = 41.5%, C = 10.9%, A = 40.2%, G = 7.5%), *T. sinensis* (T = 41.6%, C = 11.5%, A = 39.3%, G = 7.6%), and *S. sinensis* (T = 42.3%, C = 10.9%, A = 39.4%, G = 7.4%). The A + T content in these four mitogenomes ranged from 80.1% (*P. lepida*) to 81.7% (*P. conjuncta* and *S. sinensis*), as shown in Table S4. A significant level of evolutionary conservation was observed in the Limacodidae mitogenomes. This conservation was observed in various aspects, such as the A + T content of the entire mitogenome, concatenated PCGs, concatenated tRNAs, rRNAs (*rrnL* and *rrnS*), and individual elements including codon positions, as well as the *apt6*, *cox1*, and *nad1*/*2* genes. Moreover, a high degree of consistency was observed concerning the nucleotide skewness of the entire genome, concatenated PCGs, and concatenated tRNAs. Negative AT skew and GC skew were observed for the full mitogenomes of Limacodidae species other than *Monema flavescens* (AT skew = 0.013; Figure S1).

### 3.2 Analyses of protein‐coding genes and codon usage

**FIGURE 3:**
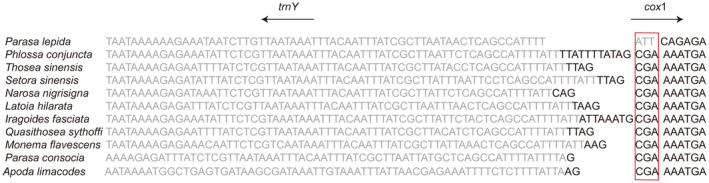
The *cox1* start codons from Limacodidae species. Genes located on the N‐strand are shown in light gray, and the *cox1* start codons are circled in red.

The total respective sizes of the 13 PCGs identified in the *P. lepida*, *P. conjuncta*, *T. sinensis*, and *S. sinensis* mitogenomes were 11,144 bp, 11,196 bp, 11,178 bp, and 11,151 bp, respectively. The combined A + T content of these 13 PCGs ranged from 78.1% (*P. lepida*) to 80% (*S. sinensis*) (Table S4). While the majority of these PCGs utilized ATN (ATT, ATG, and ATA) start codons, the *cox1* gene in the *P. conjuncta*, *T. sinensis*, and *S. sinensis* mitogenomes was found to utilize a CGA start codon. In contrast, ATT was used in *P. lepida* (Figure 3). The results of the two primer pairs sequenced by Sanger were in complete agreement with the result of high‐throughput sequencing (Figure S2).

Codon usage and relative synonymous codon usage (RSCU) were also explored for Limacodidae species, revealing identical codon preference patterns for all RSCUs. The five most commonly used codon families identified in these mitogenomes were *Leu2*, *Ile*, *Phe*, *Met*, and *Asn* (Figure S3), with UUA (*Leu2*), AUU (*Ile*), UUU (*Phe*), AUA (*Met*), and AAU (*Asn*) as the five most commonly utilized codons (Table S5). These five codons comprised 45.18% (*Parasa lepida*) – 48.68% (*Iragoides fasciata*) of all codons in the identified PCGs (Figure S4). These analyses also revealed significant AT bias in species belonging to the Limacodidae family.

### 3.3 Analyses of tRNAs and rRNAs

These four mitogenome sequences were all found to contain 22 tRNA genes, of which 14 and 8 were encoded on the J‐strand and N‐strand, respectively. The concatenated tRNA genes for these 11 Limacodidae genomes exhibited a positive AT skew and GC skew (Table S4). Except for *trnS1* (AGN), most tRNAs exhibited typical cloverleaf secondary structures (Figure S5). In addition to appropriate base pairing, some mismatched base pairs (U–U, A‐C, G‐U) were also observed in the secondary structure of these tRNAs. In these four mitogenomes, the two identified rRNA genes were separated by *trnV*, and were located between *trnL1* and the control region (CR). The length of *rrnL* ranged from 1330 bp (*P. lepida*) to 1434 bp (*T. sinensis*), while the length of *rrnS* ranged from 778 bp (*P. conjuncta*) to 785 bp (*P. lepida*) (Table 2). These rRNA genes showed significant AT bias, with A + T levels ranging from 84.2% (*P. lepida*) to 85.8% (*T. sinensis*) for *rrnL* and from 85.2% (*P. lepida*) to 86.4% (*P. conjuncta*) for *rrnS*, which aligns with previous publications on mitogenomes of other Limacodidae species (Table S4).

### 3.4 Putative control region analyses

The predicted length of the control regions (CRs) in these four mitogenomes, which were positioned between *rrnS* and *trnM*, ranged from 393 bp (*S. sinensis*) to 425 bp (*P. conjuncta*) with the A + T content ranging from 91.3% (*S. sinensis*) to 94.8% (*P. conjuncta*), which is in line with similar findings for other Limacodidae species (Table S4).

In general, four conserved CR structures were present in the mitogenomes, including (Figure S6): (1) an ATAGA motif adjacent to *rrnS* followed by a poly‐T sequence that serves as the origin of minority or light‐strand replication; (2) microsatellite‐like repeat regions (AT)n or (TA)n beginning with an ATTTA motif that is widely used for analyses of genetic diversity, kinship, and species origins; (3) a poly‐A region upstream of *trnM*; and (4) tandem repeats throughout the CR (Table S6).

### 3.5 Nucleotide diversity, evolutionary rate, and genetic distance analyses

**FIGURE 4:**
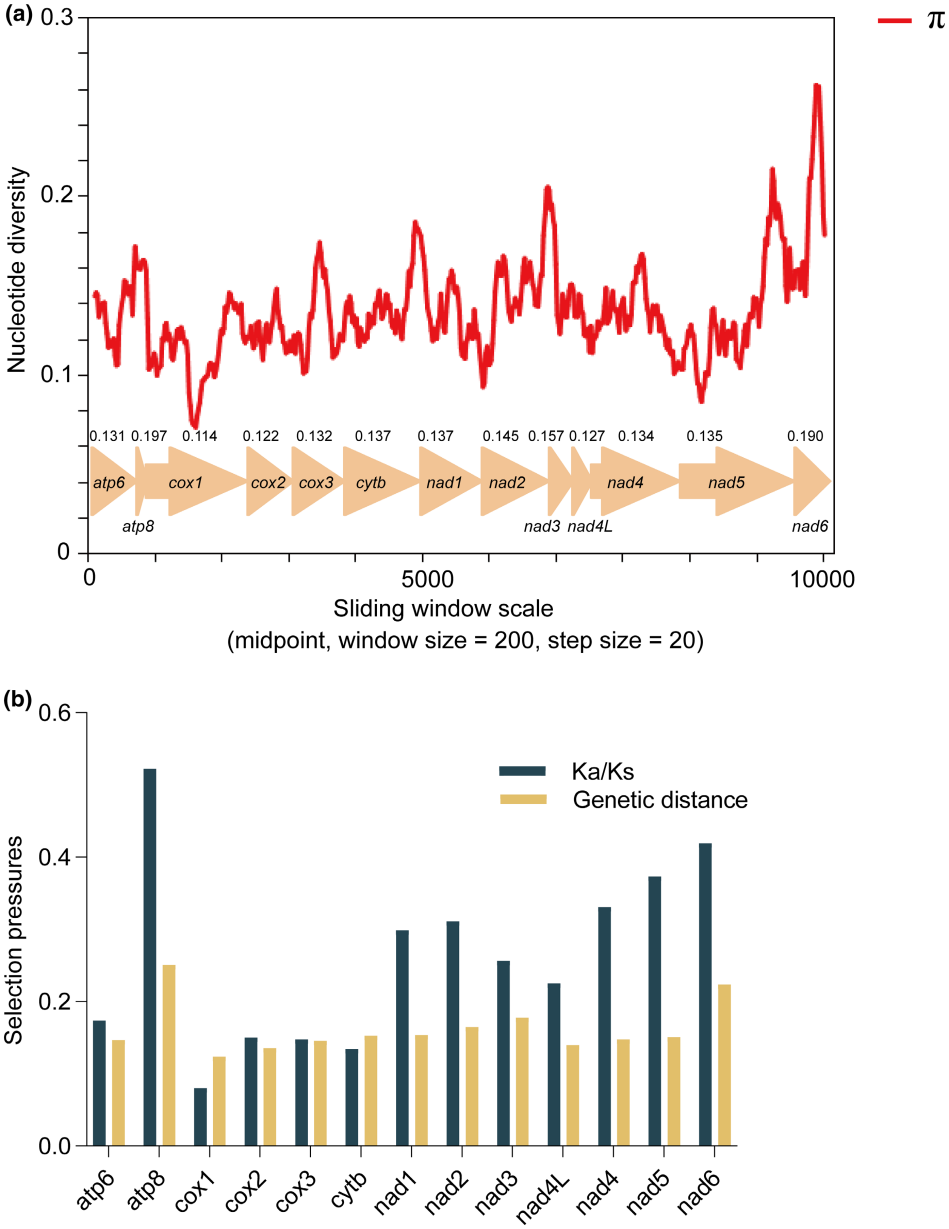
Nucleotide diversity (π) and selection pressures for 13 protein‐coding genes (PCGs) in 11 Limacodidae species. (a) Thirteen PCGs were aligned and subjected to a sliding window analysis. Average π values are indicated above the corresponding genes. (b) Nonsynonymous (Ka) to synonymous (Ks) substitution ratios and genetic distance analyses using a Kimura 2‐parameter model for these 13 PCGs from 11 Limacodidae species.

A sliding window approach was thus used to analyze the nucleotide diversity (π) for these 13 PCGs across 11 Limacodidae species (Table S4), as shown in Figure 4a. Of these 13 PCGs, *atp8* (π = 0.197) exhibited the highest degree of variability, followed by *nad6* (π = 0.19) and *nad3* (π = 0.157), with all three exhibiting relatively high nucleotide diversity values. In contrast, the nucleotide diversity observed for *cox1* (π = 0.114), *cox2* (π = 0.122), and *nad4L* (π = 0.127) was relatively limited. The Ka/Ks ratio (*ω*) values for these 13 PCGs were next calculated across these 11 Limacodidae species as a means of quantifying the corresponding evolutionary rates (Figure 4b). The values for each of the PCGs identified in these mitogenomes were less than 1, with higher values for *atp8* (*ω* = 0.522) and *nad6* (*ω* = 0.419) and the lowest values for *cox1* (*ω* = 0.08). The genetic distance values for *atp8* (0.251) and *nad6* (0.224) were higher, while *cox1* (0.124) and *cox2* (0.136) were lower (Figure 4b).

### 3.6 Phylogenetic relationships

**FIGURE 5:**
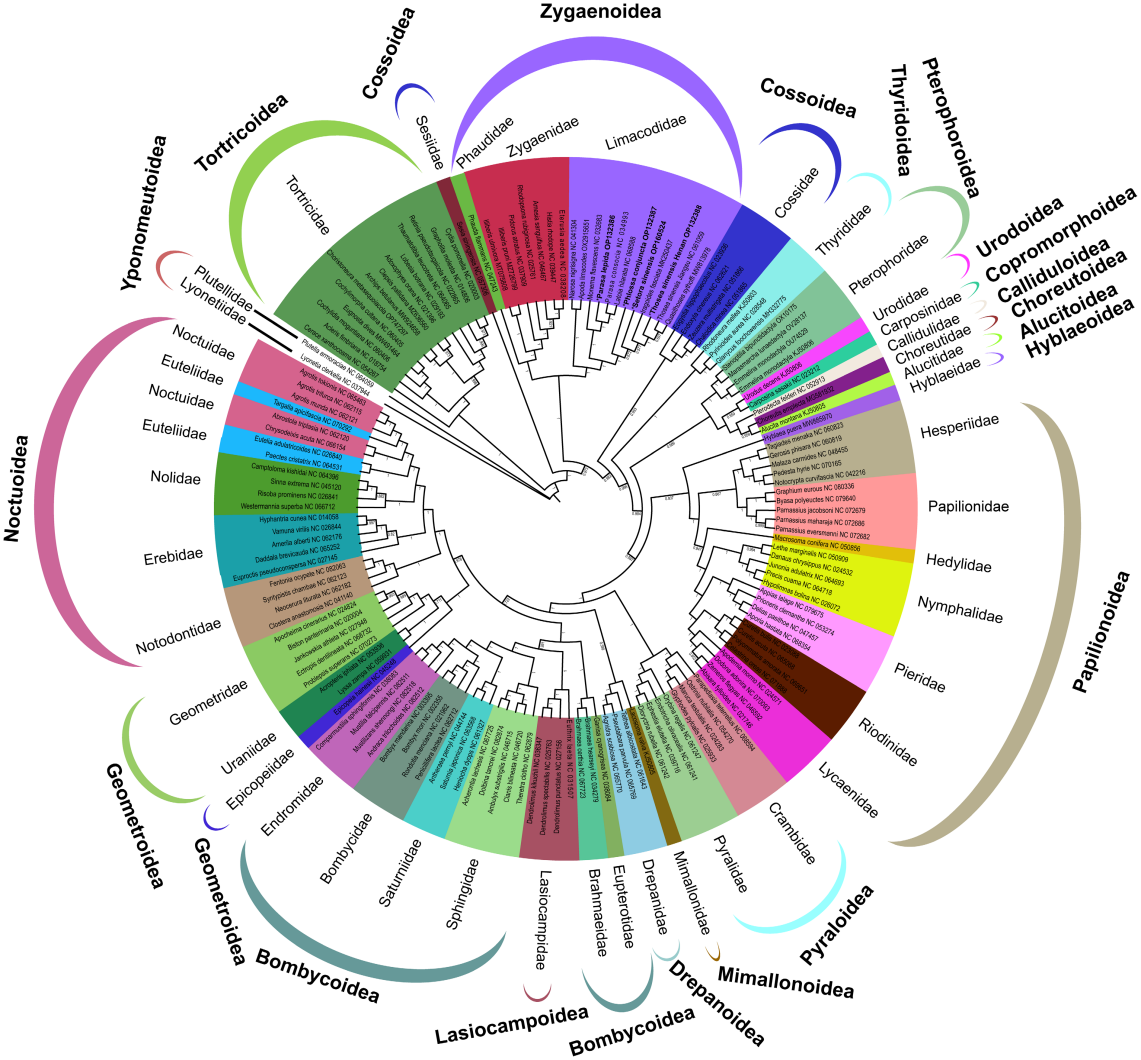
Phylogenetic relationships of 20 superfamilies of 144 Apoditrysia mitogenomes inferred from Bayesian inference (BI) using the PCGs_AA dataset. *Lyonetia clerkella* (NC_037944.1) and *Plutella armoraciae* (NC_064059.1) of the superfamily Yponomeutoidea were used as outgroups. *The sequences for species with names in bold were generated in this study.

We discussed the position of the Zygaenoidea in the clade Apoditrysia and the phylogenetic relationships within it based on the 20 superfamilies, 144 mitogenomes from 40 families, and the two Yponomeutoidea outgroup species available on NCBI. Both analysis methods (BI and ML) based on three datasets (PCGs_rRNA, PCGs_rRNA_codPosOneTwo, and PCGs_AA) produced six phylograms that showed similar topological features with high statistical support (Figure 5; Figure S7). In five trees, Sesiidae within Cossoidea formed the base of Zygaenoidea (Figure 5; Figure S7a,c–e), whereas in the BI analysis using the PCGs_rRNA dataset, Sesiidae was positioned as the base of Tortricoidea (Figure S7b). Urodoidea and Copromorphoidea were sister branches in four trees (Figure 5; Figure S7a–c), while in the other two trees, Urodoidea was placed at the base of Tortricoidea (Figure S7d,e). Here, we had chosen to show a tree with more of the same topology and high support for the Zygaenoidea (Figure 5). The overall relationship is roughly as follows: Tortricoidea + (Sesiidae + (Zygaenoidea + (Cossoidea /+Choreutidae + (others)))). The monophyly of Zygaenoidea (PP = 1, BS = 100) was strongly supported in all six phylogenetic trees (Figure 5; Figure S7). In Zygaenoidea, the family‐level relationships were determined as (Phaudidae + Zyganidae) + Limacodidae. The family Limacodidae (PP = 1, BS = 100) was recovered as monophyletic in all six phylogenetic trees. These results provide strong support for the attribution of *P. lepida*, *P. conjuncta*, *T. sinensis*, and *S. sinensis* to the Limacodidae, as well as the phylogenetic relationships within the Zygaenoidea.

## 4 DISCUSSION

In this study, four mitogenomes of *P. lepida*, *P. conjuncta*, *T. sinensis*, and *S. sinensis* were sequenced, annotated, and compared with other Limacodidae mitogenomes. By comparison, the organization and base composition of the Limacodidae mitogenomes were highly consistent, indicating the conservation of the mitogenome within the family Limacodidae. For PCG analysis, the *cox1* CGA start codon is a common finding in many of the published lepidopteran mitogenomes (Bian et al., 2020; Cameron, 2014; Djoumad et al., 2017; Jiang et al., 2022; Liu et al., 2016; Ma et al., 2016; Zhu et al., 2018). However, the ATT start codon was predicted to be utilized by the *cox1* gene of *P. lepida* in this study. To validate this, we designed two pairs of primers for Sanger sequencing and found that the results matched exactly with the high‐throughput sequencing results (Figure S7). In addition, no possible CGA codon or other ATN start codons were found near the putative start codon ATT. Thus, it is reasonable to assume that the start codon of the *cox1* gene of *P. lepida* is ATT. This is the first time this codon has been identified in the Limacodidae family (Figure 3).

Nucleotide diversity is a fundamental measure employed to evaluate genetic diversity, and it is crucial in studies centered around genetic diversity (Clark et al., 2007; Olson et al., 2010). A sliding window was used to analyze the nucleotide diversity (π) of 13 PCGs in the Limacodidae species (Figure 4a). Of these 13 PCGs, *atp8* (π = 0.197) and *nad6* (π = 0.19) exhibited the greatest degree of variability. The *ω* value is used to evaluate whether a specific gene is influenced by purifying selection (0 < *ω* < 1), neutral evolution (*ω* = 1), or positive selection (*ω* > 1) (Meiklejohn et al., 2007). The Ka/Ks ratio (*ω*) for each PCG identified in these mitogenomes was found to be less than 1, indicating that all of these genes are under purifying selection. Of these 13 PCGs, *cox1* (*ω* = 0.08) exhibited the strongest purifying selection and the lowest evolutionary rate, whereas *atp8* (*ω* = 0.522) and *nad6* (*ω* = 0.419) were subjected to relatively weak purifying selection and thus had relatively rapid evolutionary rates. Similar results were also obtained through pairwise calculations of genetic distances, with higher genetic distance values for *atp8* (0.251) and *nad6* (0.224) genes, indicating the comparatively rapid evolution of these genes. This is in stark contrast to the lower values observed for *cox1* (0.124) and *cox2* (0.136), which are consistent with slower relative evolution (Figure 4b). Thus, the *atp8* and *nad6* genes were also identified as promising candidate DNA markers that may enable species delimitation by clarifying phylogenetic relationships among slug moth species.

According to the latest phylogenetic hypothesis of Lepidoptera, Yponomeutoidea is a sister to Apoditrysia (Kawahara et al., 2019), so two Yponomeutoidea species (*L. clerkella* and *P. armoraciae*) were chosen as the outgroups in this study. Long‐branched attraction (LBA) is a major obstacle to phylogenetic reconstruction, and excluding the third codon reduces the effect of LBA (Qu et al., 2017; Sanderson et al., 2000). Therefore, in this study, two different nucleotide datasets (PCGs_rRNA and PCGs_rRNA_codPosOneTwo) and PCGs_AA datasets for phylogenetic analysis were used. The topological features of the six phylogenetic trees obtained exhibited minor variations. The majority of topological discrepancies were observed on branches with low support, possibly due to variations in the datasets used and differences between the BI and ML phylogenetic methods (Liu et al., 2021). In the future, expanding genetic data may assist in resolving this problem.

Cossoidea and Zygaenoidea were sister groups supported by some previous morphological and molecular evidence (Bao et al., 2019; Bian et al., 2020; Minet, 1991; Scott, 1986). Phylogenetic analysis of 13 PCGs based on mitogenomes showed a close phylogenetic relationship among Zygaenoidea, Papilionoidea, and Tortricidea by Liu et al. (2016). However, Cossoidea was recovered as polyphyletic, and the Sesiidae of Cossoidea were more closely related to Zygaenoidea in the present (Figure 5; Figure S7a,c–e). Their overall relationship was Tortricoidea + (Sesiidae + (Zygaenoidea + (Cossoidea/+Choreutoidea + (others)))). The phylogenetic position of Zygaenoidea in the clade Apoditrysia needs further study.

Some studies consider Phaudidae as a subfamily of Zygaenidae (Fänger, 1999; Minet, 1991). Niehuis et al. (2006) conducted a study that found Phaudidae to be closely related to Lacturidae and the sister group of Limacodidae + (Somabrachyidae + Himantopteridae). In addition, Zygaenidae was shown to be the sister group of this big branch. Zhang, Gao, et al. (2020) reported that Phaudidae is a monophyletic family sister to Zygaenidae. However, *Phauda flamman* of the Phaudidae belongs to the Zygaenidae, as determined in our study of six trees (Figure 5; Figure S7). Phaudidae may belong to a subfamily of Zygaenidae. Increasing the dataset may help to solve such problems. Within the family, Limacodidae, *Monema*, *Parasa*, and *Latoia* formed a branch, as did *Setora*, *Phlossa*, *Iragoides*, *Thosea*, and *Quasithosea*. These two sister branches and the sister clade *Narosa* and *Apoda* exhibited the strongestsupport (PP = 1, BS = 100) among the six trees constructed in this study (Figure 5; Figure S7). However, *Parasa consocia* was found to be more closely related to *Latoia hilarata* than to *P. lepida*, while *T. sinensis* Jiangsu (NC_061059.1) was more closely related to *Quasithosea sythoffi* than to *T. sinensis* Henan (OP132388) (Figure 5; Figure S7b–e). However, given the sample limitations for these analyses, the phylogenetic connections established in these studies cannot be considered definitive.

While mitogenomic sequences are commonly used when inferring phylogenetic relationships among insect species, the sole reliance on these data is nonetheless subject to certain limitations. Host mitochondrial DNA variations at the intra‐specific or inter‐specific levels can be influenced by *Wolbachia* bacteria, leading to divergence during mitogenome‐based phylogenetic analysis observed in butterflies (Jiang et al., 2018; Kodandaramaiah et al., 2013; Sucháčková Bartoňová et al., 2021; Whitworth et al., 2007). At the intra‐specific level, *Wolbachia* generates selective sweep through selective pressure, which can cause the rapid expansion of host mitochondrial haplotypes associated with *Wolbachia* through the hitch‐hike effect, thereby increasing the proportion of these mitochondrial haplotypes in the host population and consequently reducing the mitochondrial DNA diversity in the host population (Deng et al., 2021; Jiang et al., 2018; Morrow & Riegler, 2021). *Wolbachia* may lead to mitochondrial introgression between closely related species at inter‐specific levels (Gompert et al., 2008; Jackel et al., 2013). Large integrated datasets are needed in the future to improve phylogenetic resolution among lepidopteran taxa, such as greater integrated datasets of mitogenomes, nuclear genes, and morphological characters.

## 5 CONCLUSIONS

In summary, the complete mitogenomes of *P. lepida*, *P. conjuncta*, *T. sinensis*, and *S. sinensis* were sequenced in this study, enabling comparative evolutionary analyses of the mitochondrial genomes of different members of the slug moth family Limacodidae. The Limacodidae species showed a significant level of mitogenomic conservation in terms of genome size, base composition, codon use, and PCGs. The use of the ATT start codon by the *cox1* gene in *P. lepida* is the first documented use of this start codon in sequenced Limacodidae mitogenomes. The *atp8* and *nad6* genes were also identified as promising candidate DNA markers that may enable species delimitation by clarifying phylogenetic relationships among slug moth species. BI‐ and ML‐based phylogenetic analyses based on three datasets in addition provided strong support for the monophyletic origins of the Zygaenoidea. Phylogenetic analyses indicate that *P. lepida*, *P. conjuncta*, *T. sinensis*, and *S. sinensis* are members of Limacodidae.

## AUTHOR CONTRIBUTIONS

**Feng Jiang:** Conceptualization (equal); data curation (equal); formal analysis (lead); methodology (lead); resources (equal); software (lead); validation (equal); writing – original draft (lead). **Xu‐Dong Yu:** Investigation (lead). **En‐Tao Sun:** Resources (equal); supervision (lead); validation (equal); visualization (equal). **Sheng‐Li Gu:** Software (equal); supervision (equal). **Ying Liu:** Resources (equal); software (equal); visualization (equal). **Ting Liu:** Conceptualization (equal); validation (equal); writing – review and editing (lead).

## CONFLICT OF INTEREST STATEMENT

The authors declare that they have no competing interests.

### OPEN RESEARCH BADGES

This article has earned Open Data and Open Materials badges. Data and materials are available at [https://www.ncbi.nlm.nih.gov/nuccore/OP132386; https://www.ncbi.nlm.nih.gov/nuccore/OP132387; https://www.ncbi.nlm.nih.gov/nuccore/OP132388; https://www.ncbi.nlm.nih.gov/nuccore/OP160524; and https://www.ncbi.nlm.nih.gov/bioproject/PRJNA1028338].

### DATA AVAIBILITY STATEMENT

The data presented in this study can be found in GenBank under accession numbers OP132386–OP132388 and OP160524. The sequencing data in this study have been submitted to the SRA database under the project PRJNA1028338.

## Supporting information


Figure S1

Figure S2

Figure S3

Figure S4

Figure S5

Figure S6

Figure S7

Table S1

Table S2

Table S3

Table S4

Table S5

Table S6
