# Supplementary figures and images for: Mitochondrial genomes of four slug moths (Lepidoptera, Limacodidae): Genome description and phylogenetic implications
Source: Ecol Evol. 2024 Apr 29;14(5):e11319. doi: 10.1002/ece3.11319 (PMC11057057; doi:10.1002/ece3.11319)

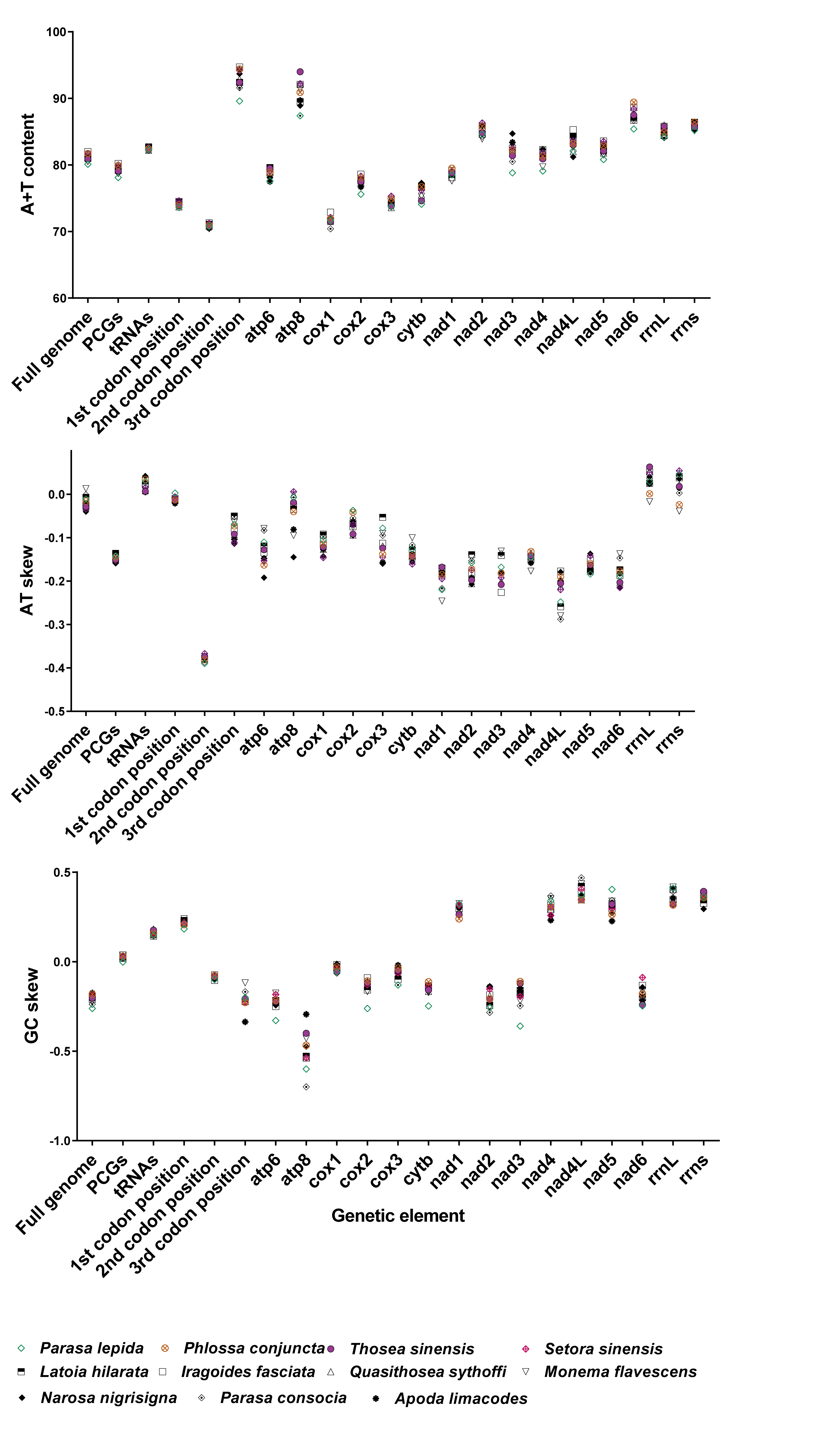

Supplement: Supplementary file 1 — Figure S1 [file ECE3-14-e11319-s001.tif]

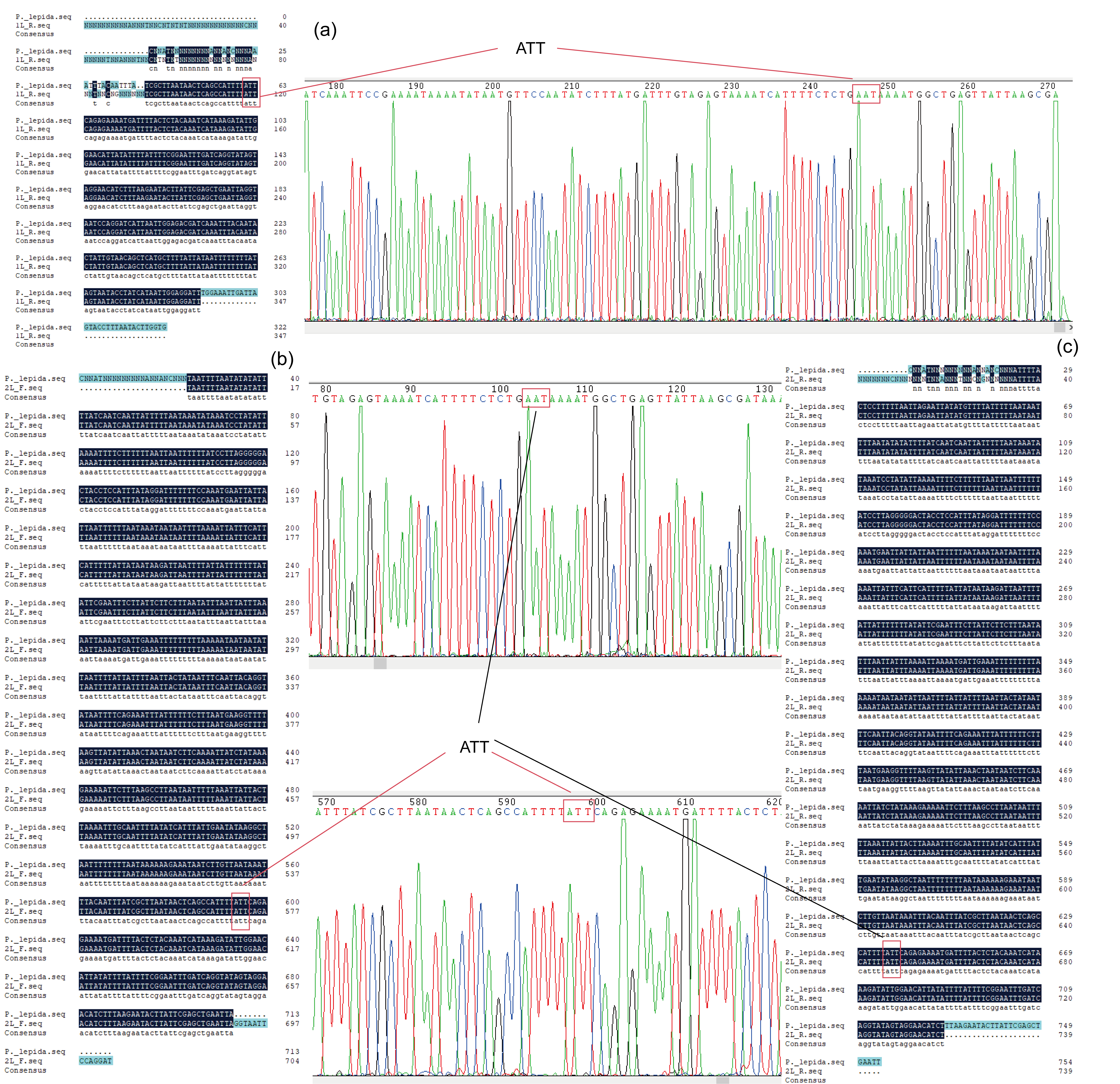

Supplement: Supplementary file 2 — Figure S2 [file ECE3-14-e11319-s002.tif]

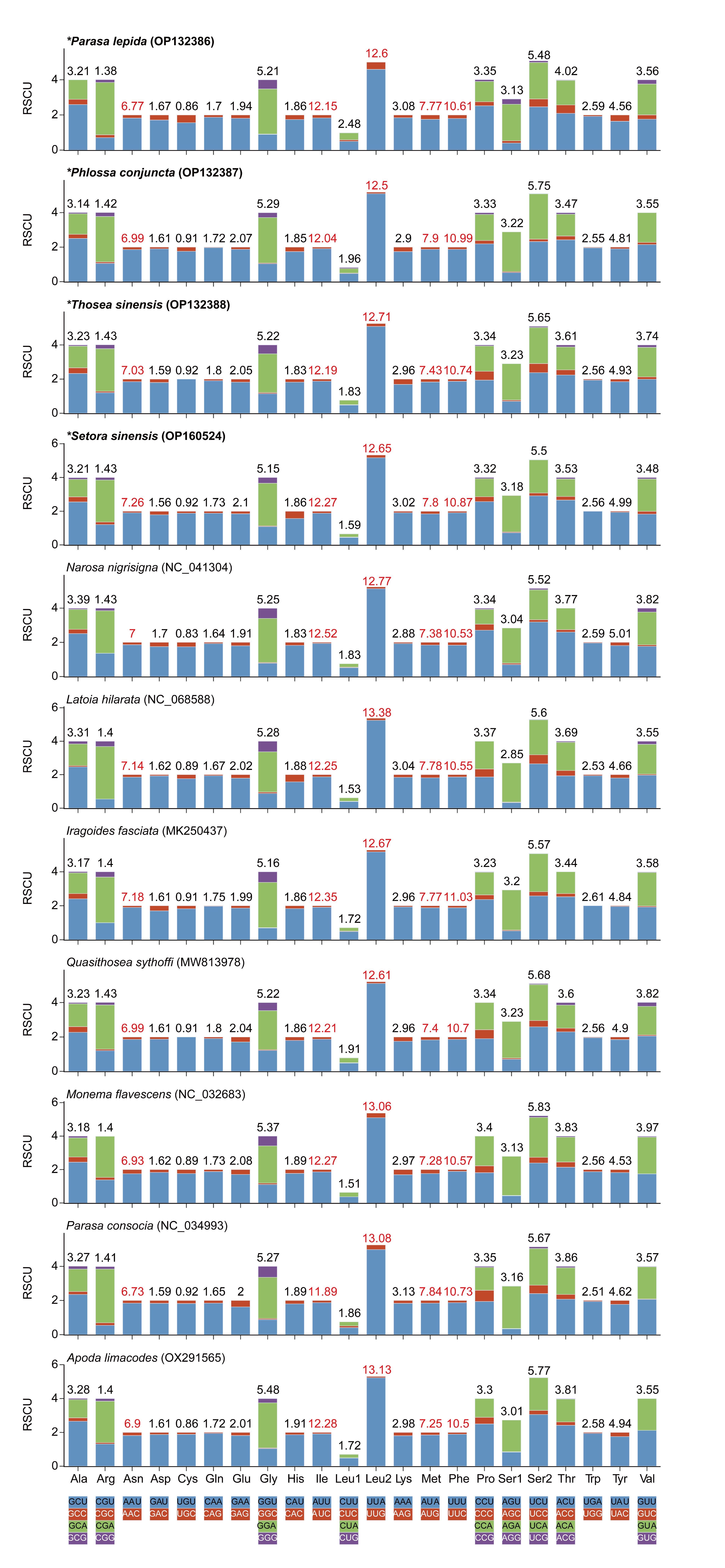

Supplement: Supplementary file 3 — Figure S3 [file ECE3-14-e11319-s003.tif]

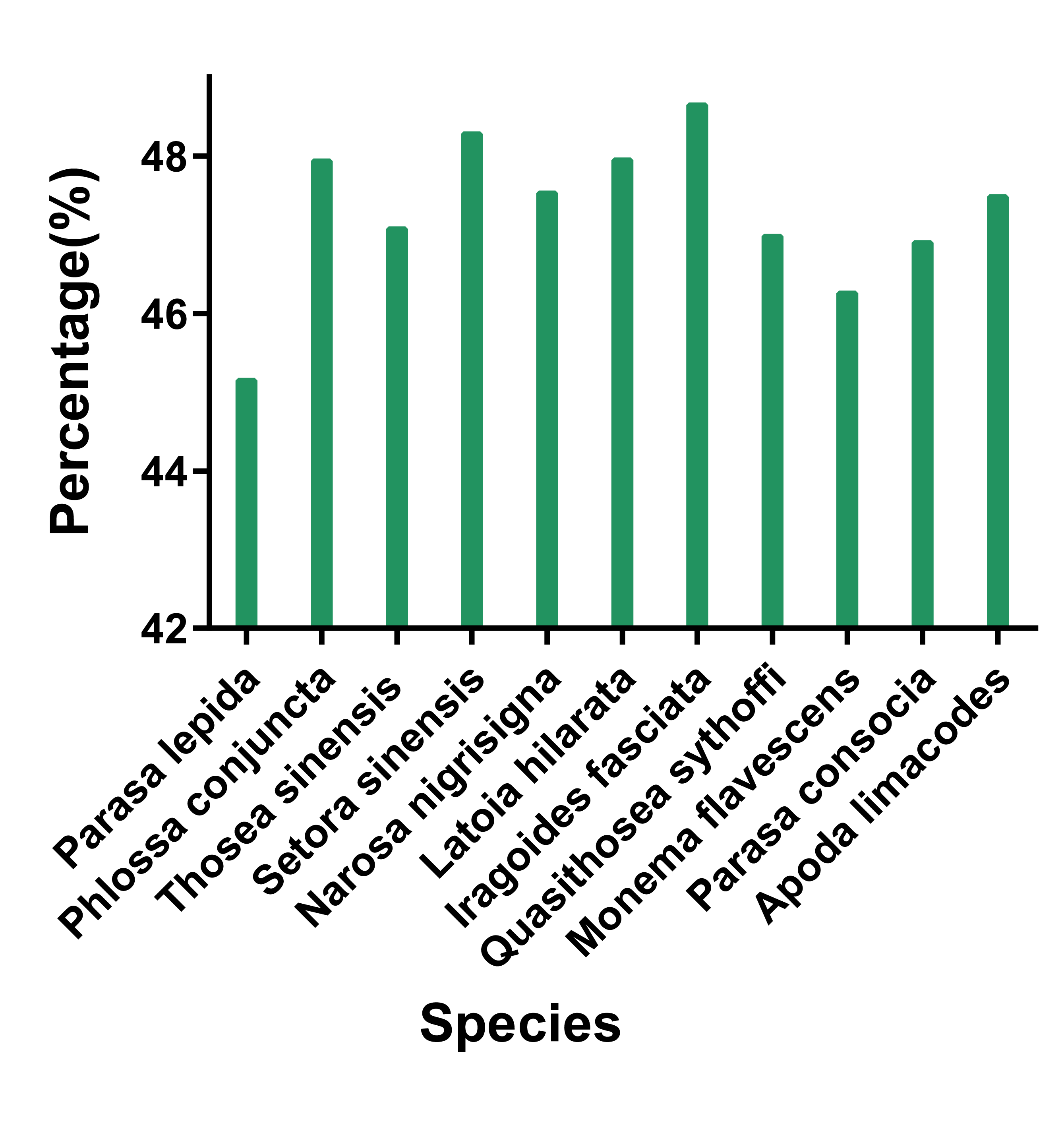

Supplement: Supplementary file 4 — Figure S4 [file ECE3-14-e11319-s008.tif]

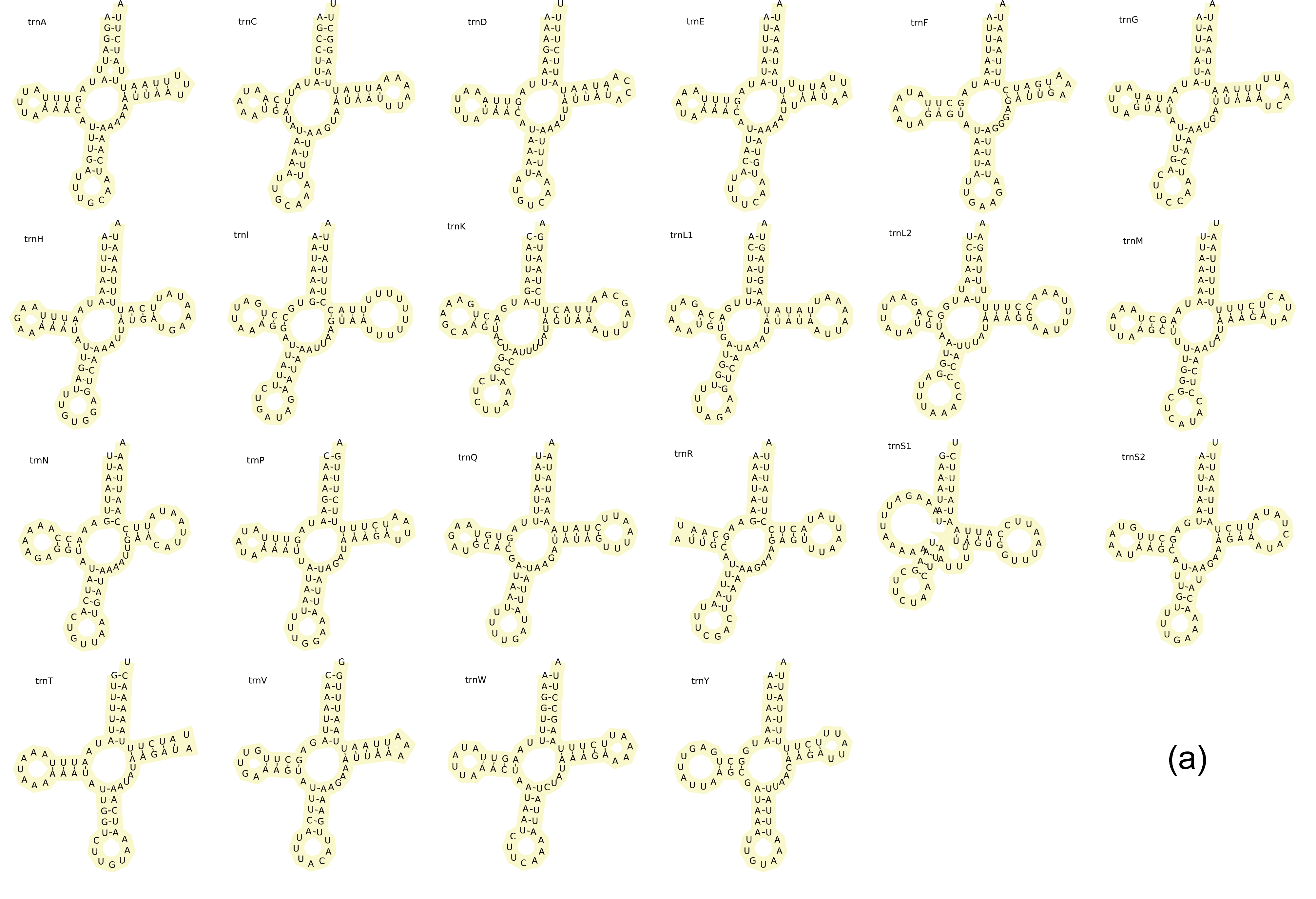

Supplement: Supplementary file 5 — Figure S5 [file ECE3-14-e11319-s005.zip › Fig S5a.tif]

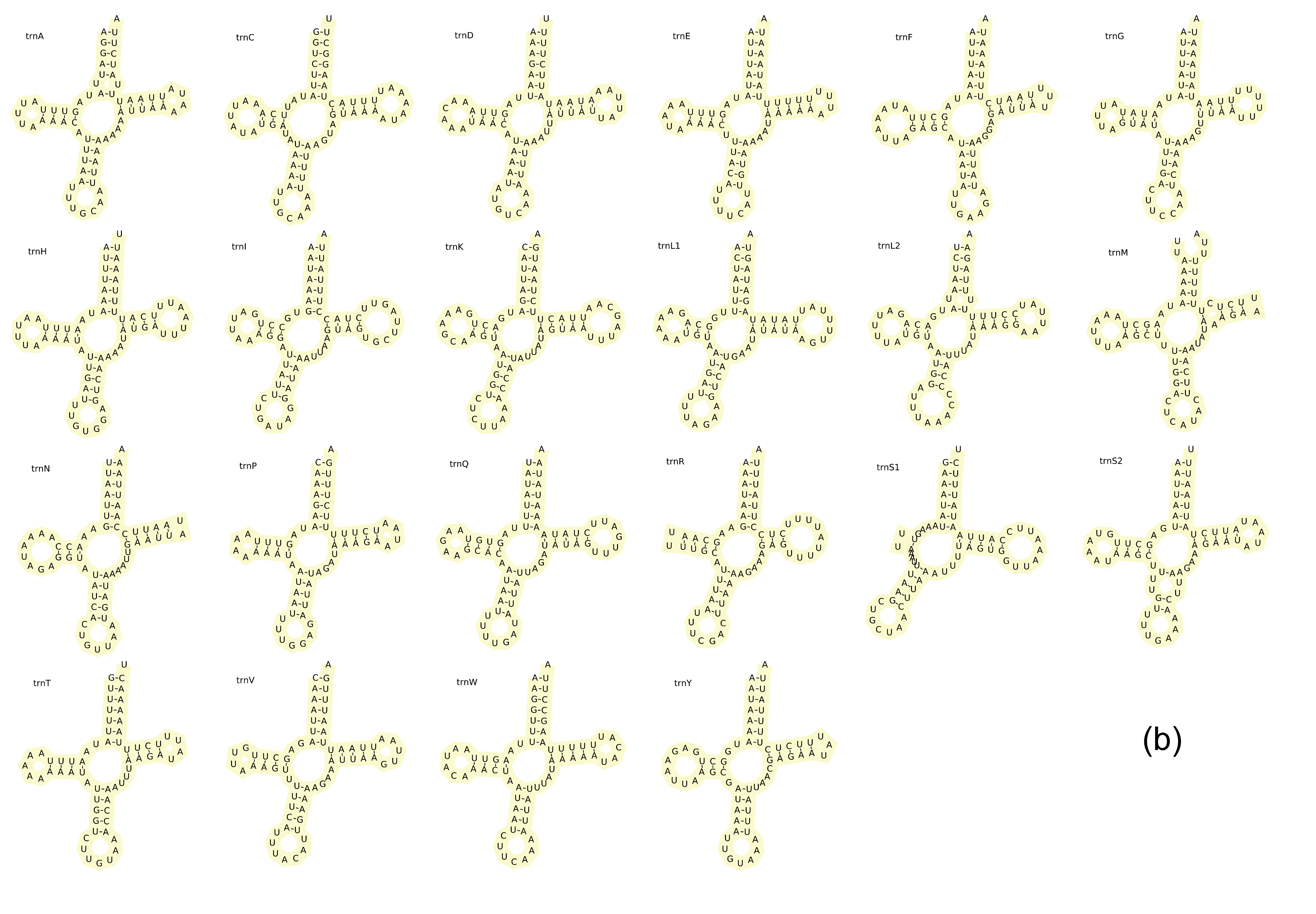

Supplement: Supplementary file 5 — Figure S5 [file ECE3-14-e11319-s005.zip › Fig S5b.tif]

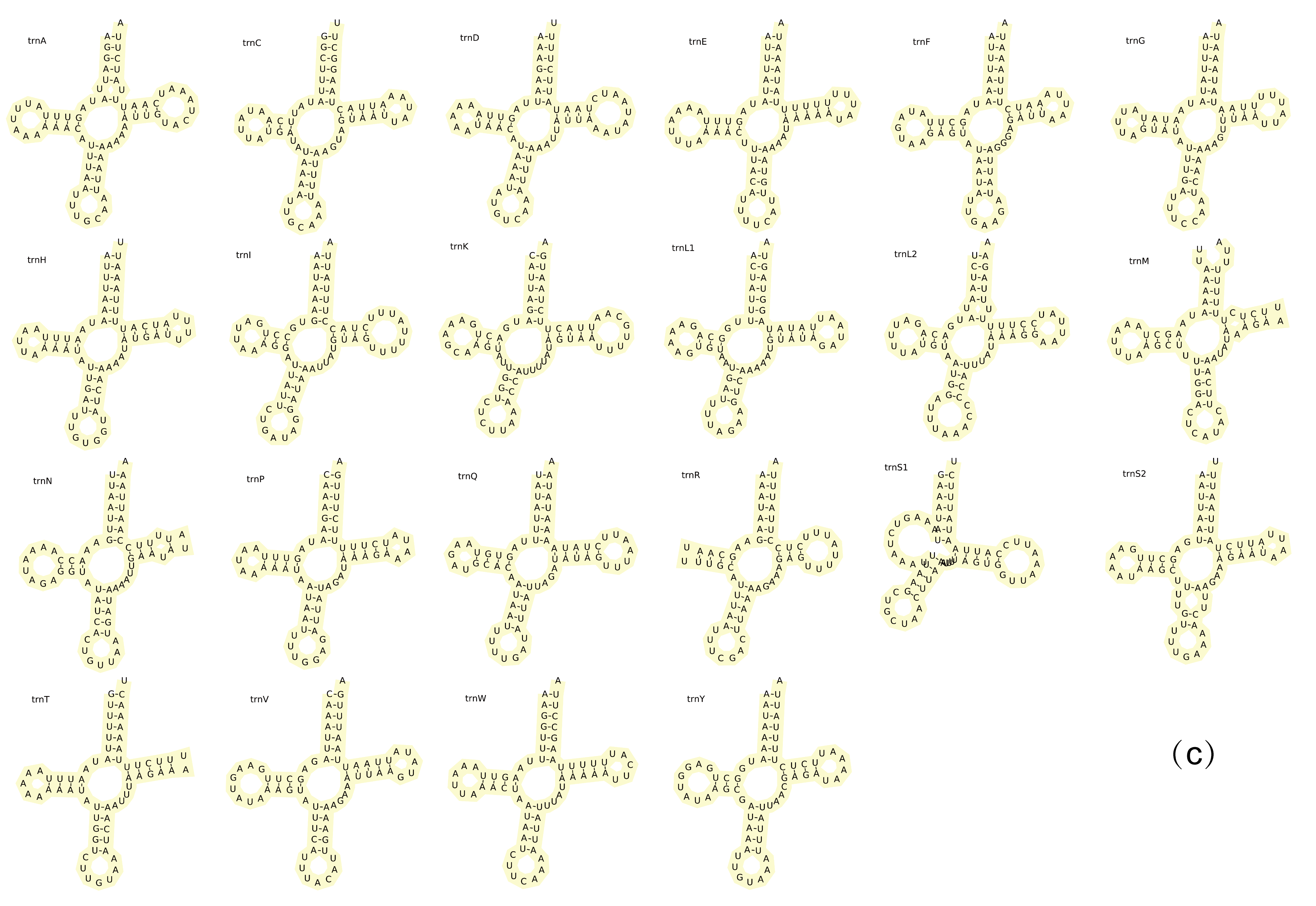

Supplement: Supplementary file 5 — Figure S5 [file ECE3-14-e11319-s005.zip › Fig S5c.tif]

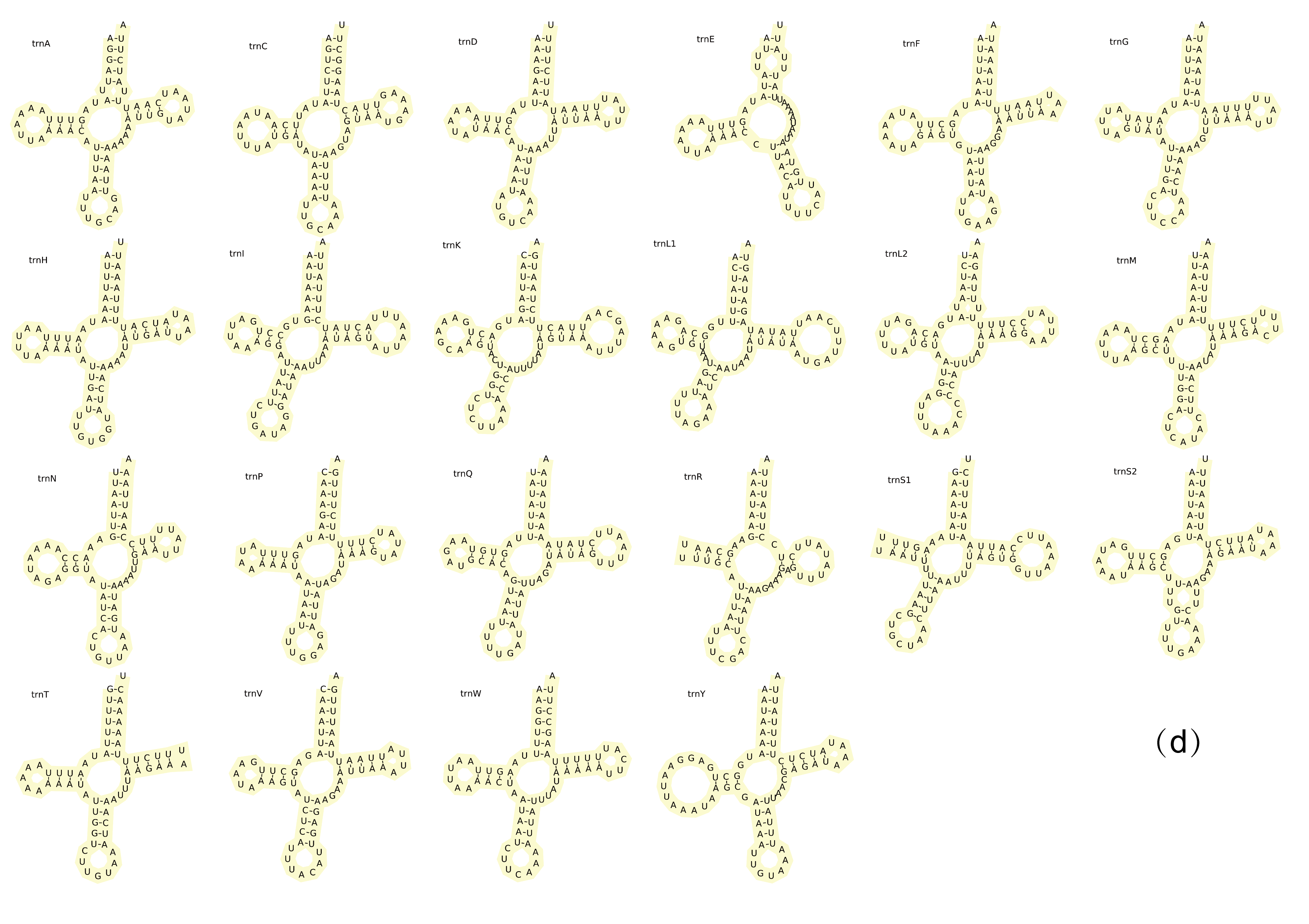

Supplement: Supplementary file 5 — Figure S5 [file ECE3-14-e11319-s005.zip › Fig S5d.tif]

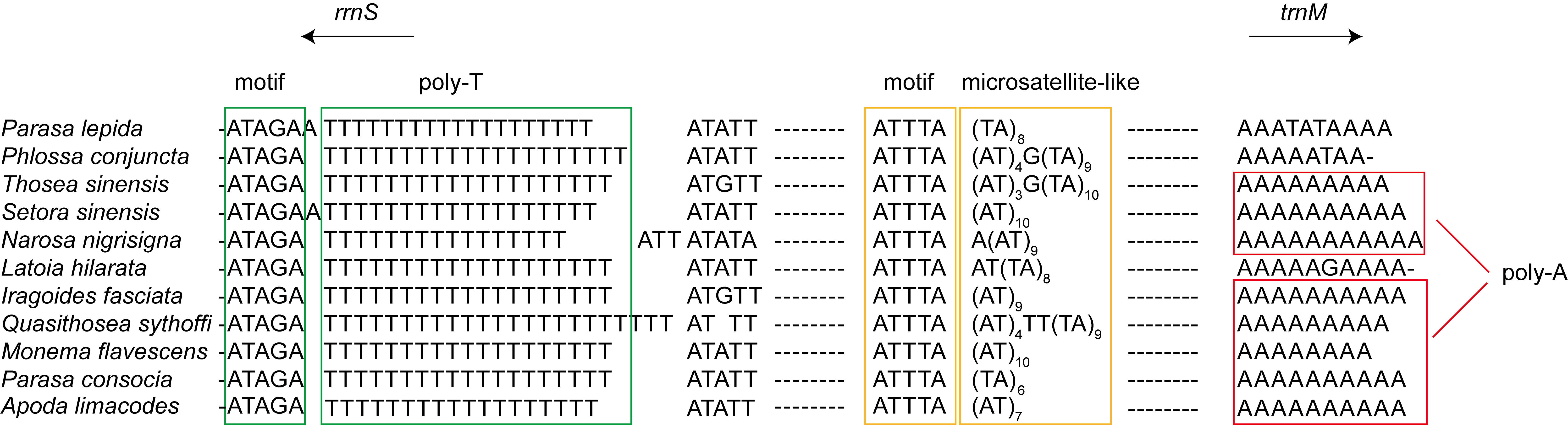

Supplement: Supplementary file 6 — Figure S6 [file ECE3-14-e11319-s011.tif]

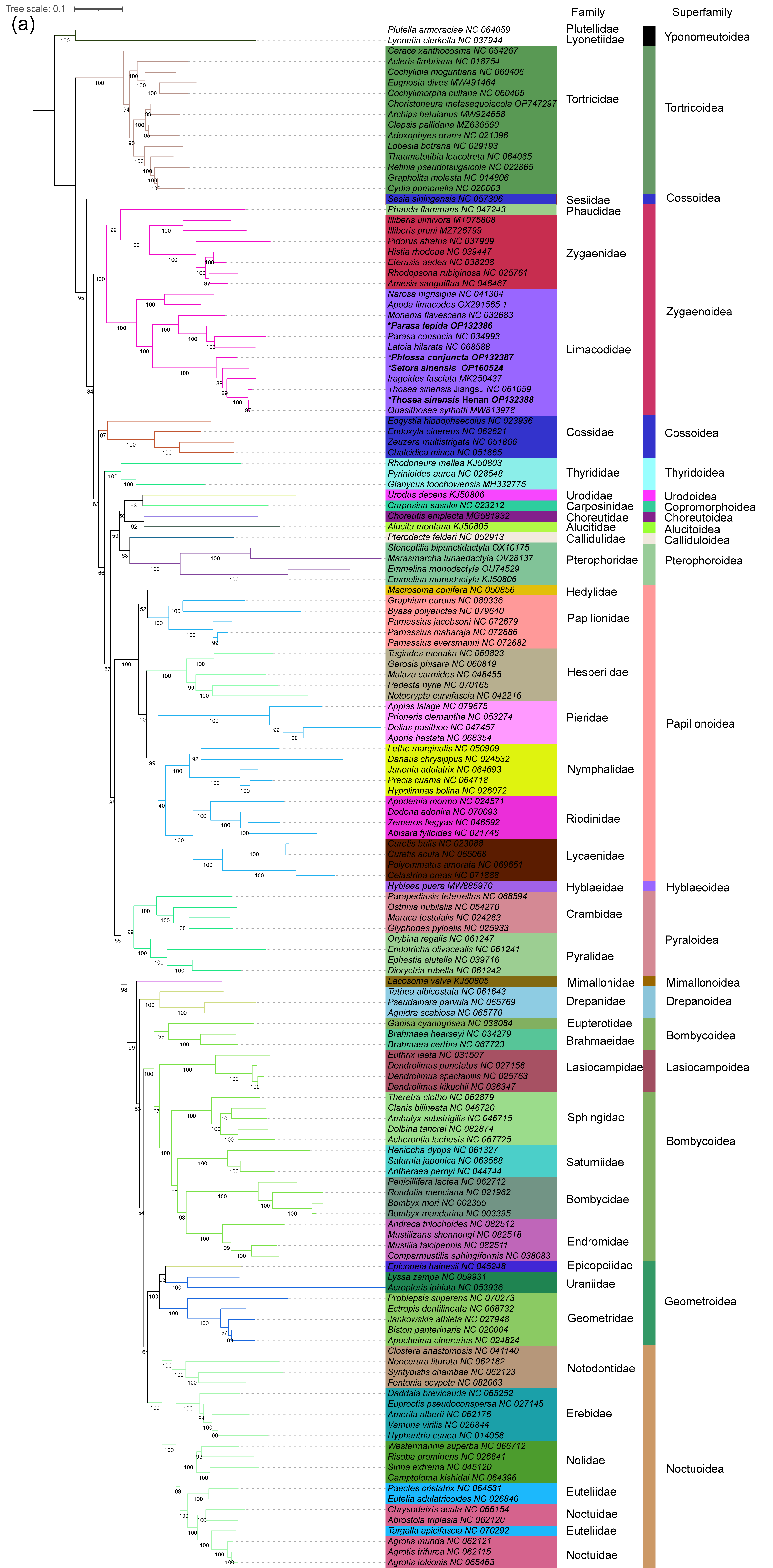

Supplement: Supplementary file 7 — Figure S7 [file ECE3-14-e11319-s004.zip › Fig S7a.tif]

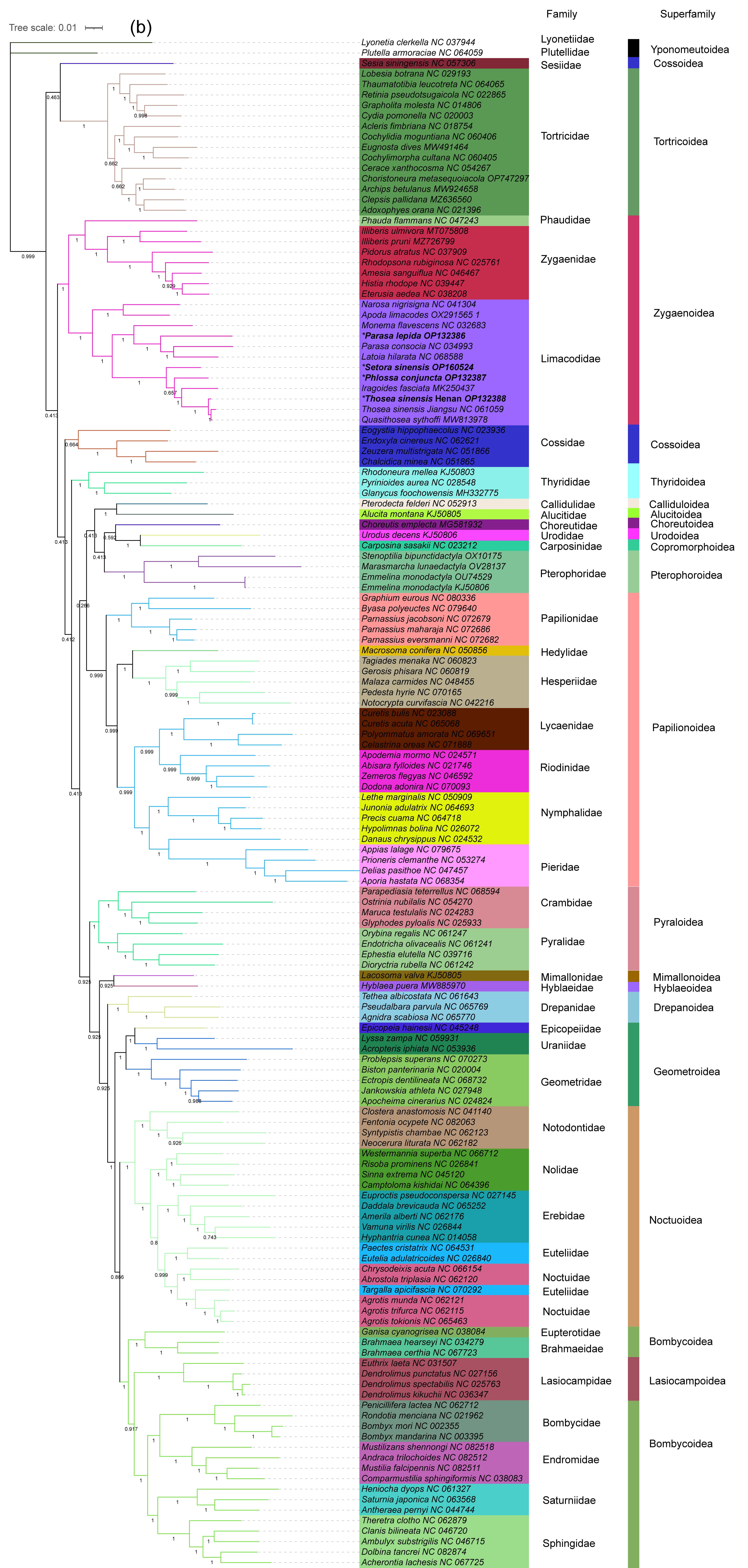

Supplement: Supplementary file 7 — Figure S7 [file ECE3-14-e11319-s004.zip › Fig S7b.tif]

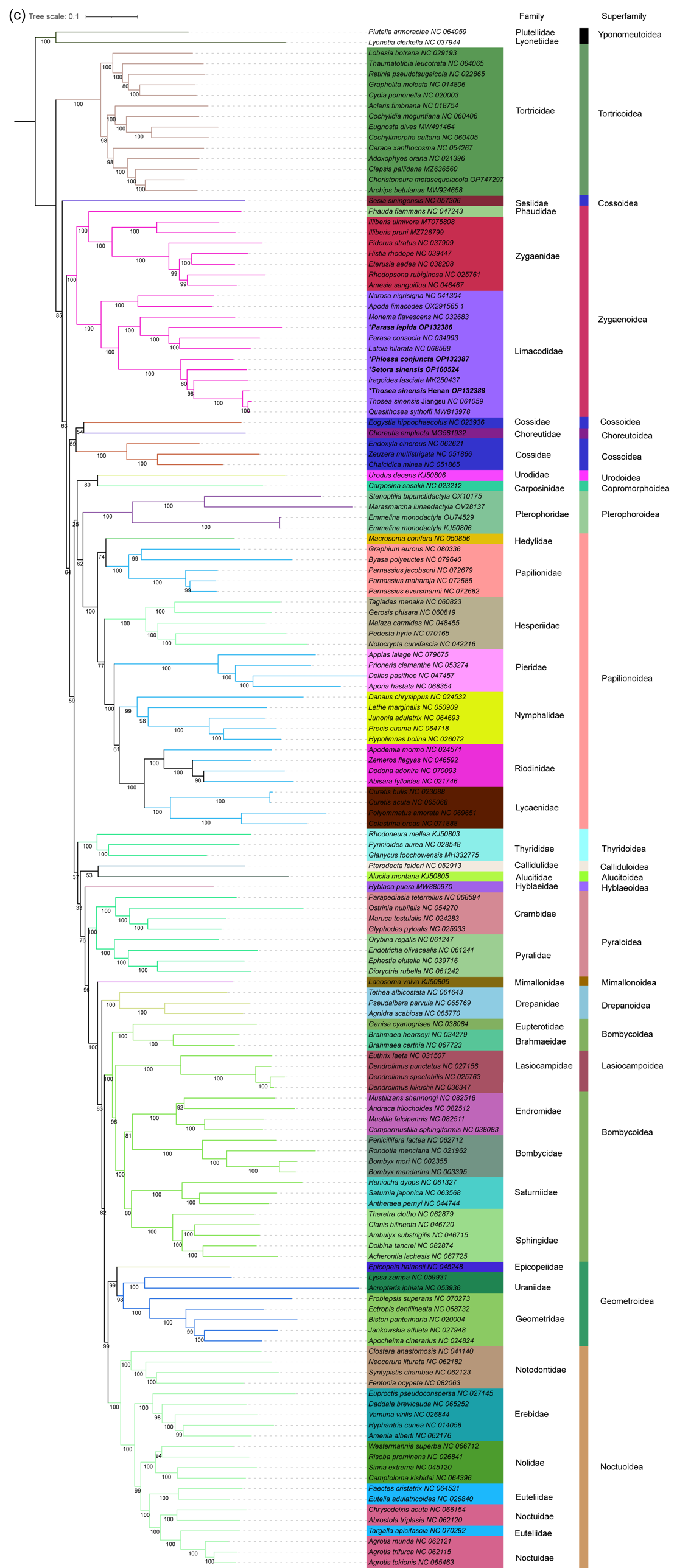

Supplement: Supplementary file 7 — Figure S7 [file ECE3-14-e11319-s004.zip › Fig S7c.tif]

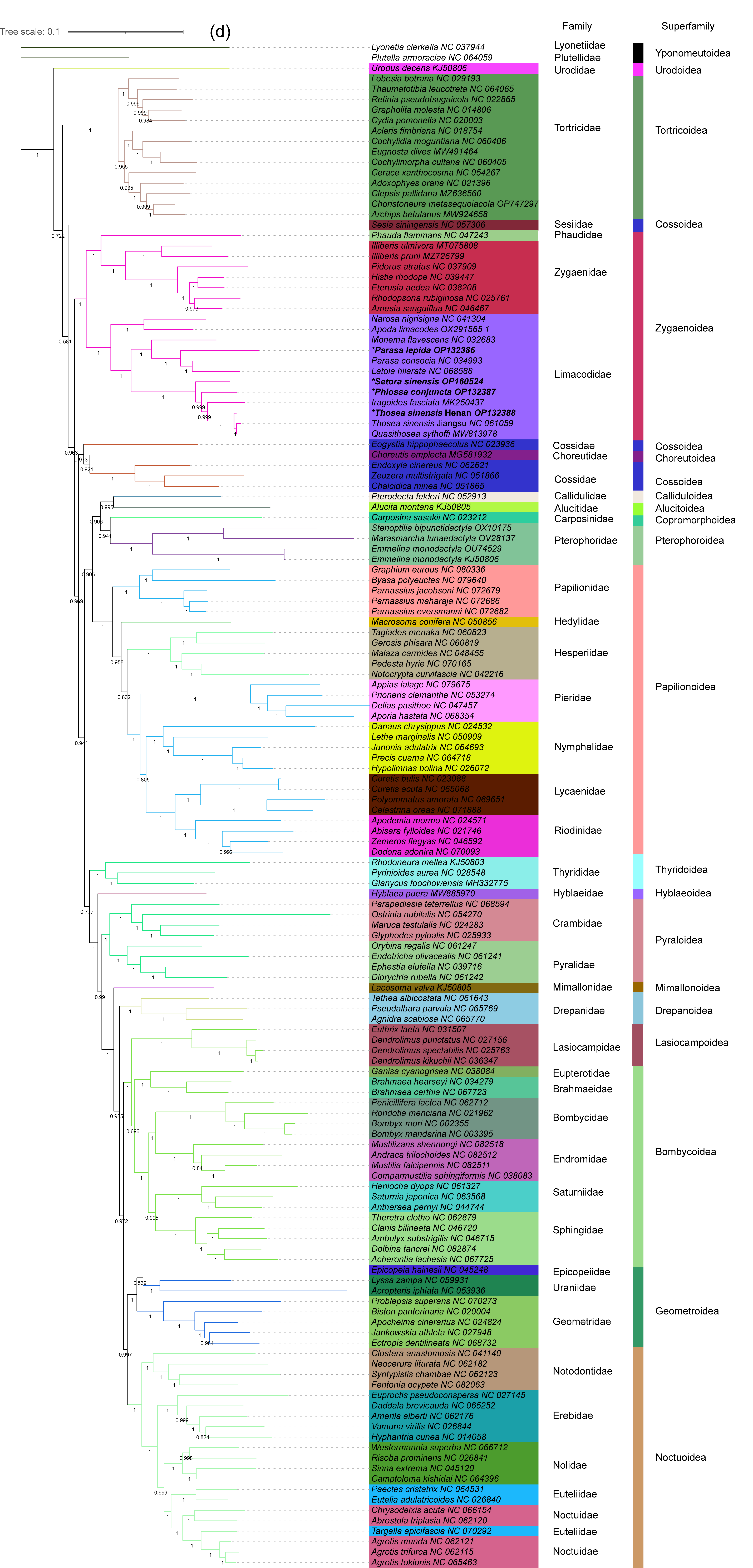

Supplement: Supplementary file 7 — Figure S7 [file ECE3-14-e11319-s004.zip › Fig S7d.tif]

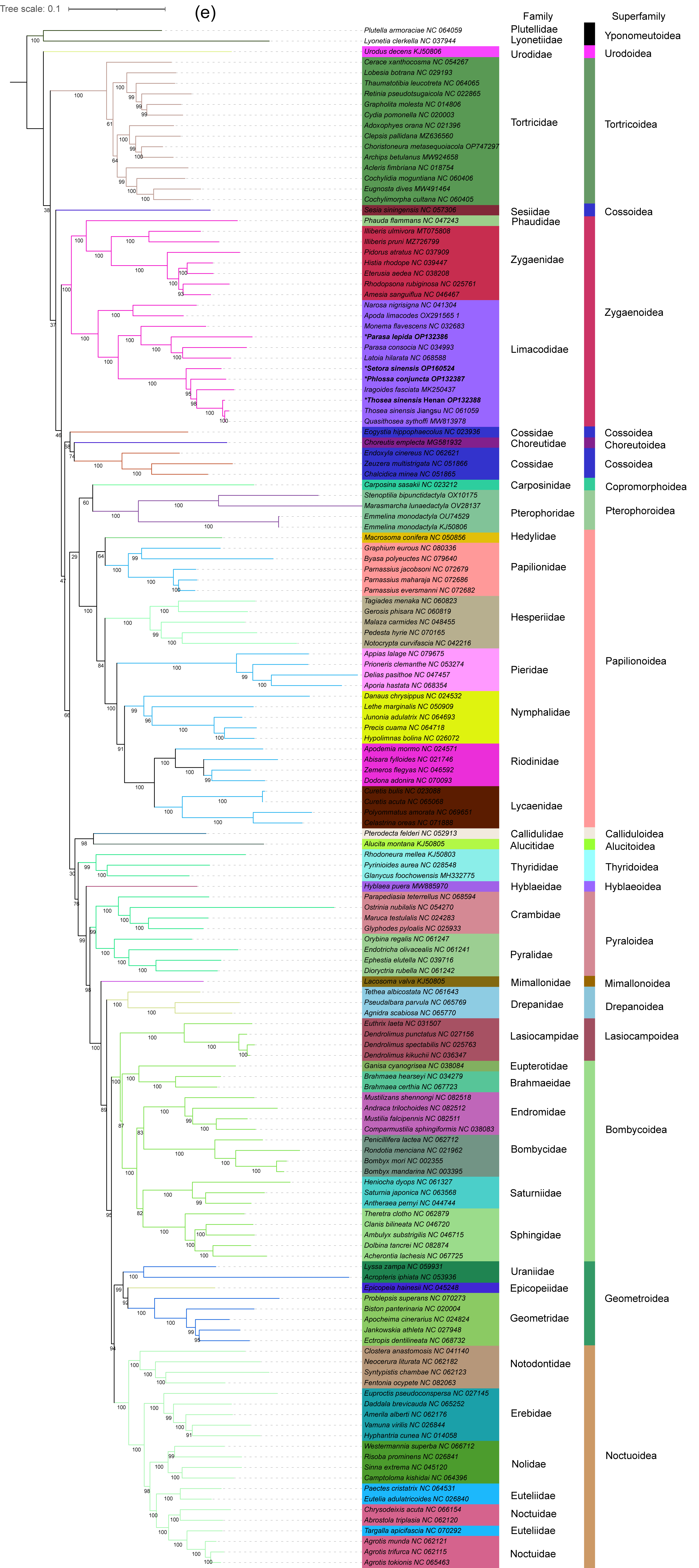

Supplement: Supplementary file 7 — Figure S7 [file ECE3-14-e11319-s004.zip › Fig S7e.tif]
