# Supplementary material for: Mitochondrial genomes of four slug moths (Lepidoptera, Limacodidae): Genome description and phylogenetic implications
Source: Ecol Evol. 2024 Apr 29;14(5):e11319. doi: 10.1002/ece3.11319 (PMC11057057; doi:10.1002/ece3.11319)
Supplement: Supplementary file 12 — Table S5 [file ECE3-14-e11319-s010.docx]

Table S5 Codon usage of protein genes family Limacodidae.

| Codon usage of protein genes in ***Parasa lepida* *** mitogenome | | | | | | | | | | | | | | | | | | | | | | |
| --- | --- | --- | --- | --- | --- | --- | --- | --- | --- | --- | --- | --- | --- | --- | --- | --- | --- | --- | --- | --- | --- | --- |
| Codon Table: 5 | | | | | | | | | | | | | | | | | | | | | | |
| Domain: Data | | | | | | | | | | | | | | | | | | | | | | |
| Codon | | Count | | RSCU | | Codon | | Count | | RSCU | Codon | | | Count | | RSCU | Codon | | | Count | | RSCU |
| UUU(F) | | 353 | | 1.8 | | UCU(S) | | 98 | | 2.46 | UAU(Y) | | | 139 | | 1.64 | UGU(C) | | | 25 | | 1.56 |
| UUC(F) | | 40 | | 0.2 | | UCC(S) | | 18 | | 0.45 | UAC(Y) | | | 30 | | 0.36 | UGC(C) | | | 7 | | 0.44 |
| UUA(L) | | 429 | | 4.6 | | UCA(S) | | 83 | | 2.08 | UAA(*) | | | 8 | | 2 | UGA(W) | | | 92 | | 1.92 |
| UUG(L) | | 38 | | 0.41 | | UCG(S) | | 4 | | 0.1 | UAG(*) | | | 0 | | 0 | UGG(W) | | | 4 | | 0.08 |
| CUU(L) | | 47 | | 0.5 | | CCU(P) | | 78 | | 2.52 | CAU(H) | | | 60 | | 1.74 | CGU(R) | | | 9 | | 0.71 |
| CUC(L) | | 7 | | 0.08 | | CCC(P) | | 7 | | 0.23 | CAC(H) | | | 9 | | 0.26 | CGC(R) | | | 2 | | 0.16 |
| CUA(L) | | 37 | | 0.4 | | CCA(P) | | 36 | | 1.16 | CAA(Q) | | | 59 | | 1.87 | CGA(R) | | | 38 | | 2.98 |
| CUG(L) | | 1 | | 0.01 | | CCG(P) | | 3 | | 0.1 | CAG(Q) | | | 4 | | 0.13 | CGG(R) | | | 2 | | 0.16 |
| AUU(I) | | 412 | | 1.83 | | ACU(T) | | 78 | | 2.09 | AAU(N) | | | 228 | | 1.82 | AGU(S) | | | 16 | | 0.4 |
| AUC(I) | | 38 | | 0.17 | | ACC(T) | | 18 | | 0.48 | AAC(N) | | | 23 | | 0.18 | AGC(S) | | | 5 | | 0.13 |
| AUA(M) | | 252 | | 1.75 | | ACA(T) | | 52 | | 1.4 | AAA(K) | | | 105 | | 1.84 | AGA(S) | | | 83 | | 2.08 |
| AUG(M) | | 36 | | 0.25 | | ACG(T) | | 1 | | 0.03 | AAG(K) | | | 9 | | 0.16 | AGG(S) | | | 12 | | 0.3 |
| GUU(V) | | 58 | | 1.76 | | GCU(A) | | 77 | | 2.59 | GAU(D) | | | 53 | | 1.71 | GGU(G) | | | 43 | | 0.89 |
| GUC(V) | | 8 | | 0.24 | | GCC(A) | | 9 | | 0.3 | GAC(D) | | | 9 | | 0.29 | GGC(G) | | | 1 | | 0.02 |
| GUA(V) | | 58 | | 1.76 | | GCA(A) | | 33 | | 1.11 | GAA(E) | | | 65 | | 1.81 | GGA(G) | | | 124 | | 2.57 |
| GUG(V) | | 8 | | 0.24 | | GCG(A) | | 0 | | 0 | GAG(E) | | | 7 | | 0.19 | GGG(G) | | | 25 | | 0.52 |
| Average# codons=3713 | | | | | | | | | | | | | | | | | | | | | | |
|  | | | | | | | | | | | | | | | | | | | | | | |
| Codon usage of protein genes in ***Phlossa conjuncta* *** mitogenome | | | | | | | | | | | | | | | | | | | | | | |
| Codon Table: 5 | | | | | | | | | | | | | | | | | | | | | | |
| Domain: Data | | | | | | | | | | | | | | | | | | | | | | |
| Codon | Count | | RSCU | | Codon | | Count | | RSCU | | | Codon | Count | | RSCU | | | Codon | Count | | RSCU | |
| UUU(F) | 383 | | 1.87 | | UCU(S) | | 97 | | 2.32 | | | UAU(Y) | 170 | | 1.9 | | | UGU(C) | 30 | | 1.76 | |
| UUC(F) | 26 | | 0.13 | | UCC(S) | | 6 | | 0.14 | | | UAC(Y) | 9 | | 0.1 | | | UGC(C) | 4 | | 0.24 | |
| UUA(L) | 457 | | 5.1 | | UCA(S) | | 110 | | 2.63 | | | UAA(*) | 10 | | 2 | | | UGA(W) | 93 | | 1.96 | |
| UUG(L) | 8 | | 0.09 | | UCG(S) | | 1 | | 0.02 | | | UAG(*) | 0 | | 0 | | | UGG(W) | 2 | | 0.04 | |
| CUU(L) | 42 | | 0.47 | | CCU(P) | | 68 | | 2.19 | | | CAU(H) | 60 | | 1.74 | | | CGU(R) | 14 | | 1.06 | |
| CUC(L) | 3 | | 0.03 | | CCC(P) | | 6 | | 0.19 | | | CAC(H) | 9 | | 0.26 | | | CGC(R) | 1 | | 0.08 | |
| CUA(L) | 23 | | 0.26 | | CCA(P) | | 47 | | 1.52 | | | CAA(Q) | 63 | | 1.97 | | | CGA(R) | 35 | | 2.64 | |
| CUG(L) | 5 | | 0.06 | | CCG(P) | | 3 | | 0.1 | | | CAG(Q) | 1 | | 0.03 | | | CGG(R) | 3 | | 0.23 | |
| AUU(I) | 428 | | 1.91 | | ACU(T) | | 78 | | 2.42 | | | AAU(N) | 242 | | 1.86 | | | AGU(S) | 22 | | 0.53 | |
| AUC(I) | 20 | | 0.09 | | ACC(T) | | 7 | | 0.22 | | | AAC(N) | 18 | | 0.14 | | | AGC(S) | 2 | | 0.05 | |
| AUA(M) | 275 | | 1.87 | | ACA(T) | | 41 | | 1.27 | | | AAA(K) | 94 | | 1.74 | | | AGA(S) | 96 | | 2.3 | |
| AUG(M) | 19 | | 0.13 | | ACG(T) | | 3 | | 0.09 | | | AAG(K) | 14 | | 0.26 | | | AGG(S) | 0 | | 0 | |
| GUU(V) | 71 | | 2.15 | | GCU(A) | | 73 | | 2.5 | | | GAU(D) | 57 | | 1.9 | | | GGU(G) | 51 | | 1.04 | |
| GUC(V) | 4 | | 0.12 | | GCC(A) | | 7 | | 0.24 | | | GAC(D) | 3 | | 0.1 | | | GGC(G) | 2 | | 0.04 | |
| GUA(V) | 57 | | 1.73 | | GCA(A) | | 35 | | 1.2 | | | GAA(E) | 72 | | 1.87 | | | GGA(G) | 130 | | 2.64 | |
| GUG(V) | 0 | | 0 | | GCG(A) | | 2 | | 0.07 | | | GAG(E) | 5 | | 0.13 | | | GGG(G) | 14 | | 0.28 | |
| Average# codons=3731 | | | | | | | | | | | | | | | | | | | | | | |
|  | | | | | | | | | | | | | | | | | | | | | | |
| Codon usage of protein genes in ***Thosea sinensis* *** mitogenome | | | | | | | | | | | | | | | | | | | | | | |
| Codon Table: 5 | | | | | | | | | | | | | | | | | | | | | | |
| Domain: Data | | | | | | | | | | | | | | | | | | | | | | |
| Codon | Count | | RSCU | | Codon | | Count | | RSCU | | | Codon | Count | | RSCU | | | Codon | Count | | RSCU | |
| UUU(F) | 373 | | 1.87 | | UCU(S) | | 98 | | 2.38 | | | UAU(Y) | 169 | | 1.85 | | | UGU(C) | 34 | | 2 | |
| UUC(F) | 26 | | 0.13 | | UCC(S) | | 22 | | 0.53 | | | UAC(Y) | 14 | | 0.15 | | | UGC(C) | 0 | | 0 | |
| UUA(L) | 457 | | 5.08 | | UCA(S) | | 87 | | 2.11 | | | UAA(*) | 10 | | 2 | | | UGA(W) | 92 | | 1.94 | |
| UUG(L) | 15 | | 0.17 | | UCG(S) | | 3 | | 0.07 | | | UAG(*) | 0 | | 0 | | | UGG(W) | 3 | | 0.06 | |
| CUU(L) | 43 | | 0.48 | | CCU(P) | | 60 | | 1.94 | | | CAU(H) | 62 | | 1.82 | | | CGU(R) | 16 | | 1.21 | |
| CUC(L) | 2 | | 0.02 | | CCC(P) | | 16 | | 0.52 | | | CAC(H) | 6 | | 0.18 | | | CGC(R) | 1 | | 0.08 | |
| CUA(L) | 23 | | 0.26 | | CCA(P) | | 46 | | 1.48 | | | CAA(Q) | 64 | | 1.91 | | | CGA(R) | 33 | | 2.49 | |
| CUG(L) | 0 | | 0 | | CCG(P) | | 2 | | 0.06 | | | CAG(Q) | 3 | | 0.09 | | | CGG(R) | 3 | | 0.23 | |
| AUU(I) | 425 | | 1.88 | | ACU(T) | | 75 | | 2.24 | | | AAU(N) | 242 | | 1.85 | | | AGU(S) | 29 | | 0.7 | |
| AUC(I) | 28 | | 0.12 | | ACC(T) | | 10 | | 0.3 | | | AAC(N) | 19 | | 0.15 | | | AGC(S) | 3 | | 0.07 | |
| AUA(M) | 253 | | 1.83 | | ACA(T) | | 45 | | 1.34 | | | AAA(K) | 93 | | 1.69 | | | AGA(S) | 88 | | 2.13 | |
| AUG(M) | 23 | | 0.17 | | ACG(T) | | 4 | | 0.12 | | | AAG(K) | 17 | | 0.31 | | | AGG(S) | 0 | | 0 | |
| GUU(V) | 69 | | 1.99 | | GCU(A) | | 70 | | 2.33 | | | GAU(D) | 53 | | 1.8 | | | GGU(G) | 56 | | 1.15 | |
| GUC(V) | 5 | | 0.14 | | GCC(A) | | 10 | | 0.33 | | | GAC(D) | 6 | | 0.2 | | | GGC(G) | 3 | | 0.06 | |
| GUA(V) | 60 | | 1.73 | | GCA(A) | | 38 | | 1.27 | | | GAA(E) | 69 | | 1.82 | | | GGA(G) | 110 | | 2.27 | |
| GUG(V) | 5 | | 0.14 | | GCG(A) | | 2 | | 0.07 | | | GAG(E) | 7 | | 0.18 | | | GGG(G) | 25 | | 0.52 | |
| Average# codons=3725 | | | | | | | | | | | | | | | | | | | | | | |
|  | | | | | | | | | | | | | | | | | | | | | | |
| Codon usage of protein genes in ***Setora sinensis* *** mitogenome | | | | | | | | | | | | | | | | | | | | | | |
| Codon Table: 5 | | | | | | | | | | | | | | | | | | | | | | |
| Domain: Data | | | | | | | | | | | | | | | | | | | | | | |
| Codon | Count | | RSCU | | Codon | | Count | | RSCU | | | Codon | Count | | RSCU | | | Codon | Count | | RSCU | |
| UUU(F) | 385 | | 1.91 | | UCU(S) | | 117 | | 2.91 | | | UAU(Y) | 179 | | 1.94 | | | UGU(C) | 32 | | 1.88 | |
| UUC(F) | 18 | | 0.09 | | UCC(S) | | 7 | | 0.17 | | | UAC(Y) | 6 | | 0.06 | | | UGC(C) | 2 | | 0.12 | |
| UUA(L) | 455 | | 5.17 | | UCA(S) | | 79 | | 1.96 | | | UAA(*) | 8 | | 2 | | | UGA(W) | 95 | | 2 | |
| UUG(L) | 14 | | 0.16 | | UCG(S) | | 1 | | 0.02 | | | UAG(*) | 0 | | 0 | | | UGG(W) | 0 | | 0 | |
| CUU(L) | 39 | | 0.44 | | CCU(P) | | 79 | | 2.57 | | | CAU(H) | 54 | | 1.57 | | | CGU(R) | 16 | | 1.21 | |
| CUC(L) | 2 | | 0.02 | | CCC(P) | | 9 | | 0.29 | | | CAC(H) | 15 | | 0.43 | | | CGC(R) | 2 | | 0.15 | |
| CUA(L) | 17 | | 0.19 | | CCA(P) | | 33 | | 1.07 | | | CAA(Q) | 60 | | 1.88 | | | CGA(R) | 33 | | 2.49 | |
| CUG(L) | 1 | | 0.01 | | CCG(P) | | 2 | | 0.07 | | | CAG(Q) | 4 | | 0.12 | | | CGG(R) | 2 | | 0.15 | |
| AUU(I) | 428 | | 1.88 | | ACU(T) | | 87 | | 2.66 | | | AAU(N) | 257 | | 1.91 | | | AGU(S) | 29 | | 0.72 | |
| AUC(I) | 27 | | 0.12 | | ACC(T) | | 7 | | 0.21 | | | AAC(N) | 12 | | 0.09 | | | AGC(S) | 2 | | 0.05 | |
| AUA(M) | 266 | | 1.84 | | ACA(T) | | 34 | | 1.04 | | | AAA(K) | 107 | | 1.91 | | | AGA(S) | 87 | | 2.16 | |
| AUG(M) | 23 | | 0.16 | | ACG(T) | | 3 | | 0.09 | | | AAG(K) | 5 | | 0.09 | | | AGG(S) | 0 | | 0 | |
| GUU(V) | 59 | | 1.83 | | GCU(A) | | 76 | | 2.55 | | | GAU(D) | 52 | | 1.79 | | | GGU(G) | 52 | | 1.09 | |
| GUC(V) | 5 | | 0.16 | | GCC(A) | | 9 | | 0.3 | | | GAC(D) | 6 | | 0.21 | | | GGC(G) | 2 | | 0.04 | |
| GUA(V) | 62 | | 1.92 | | GCA(A) | | 31 | | 1.04 | | | GAA(E) | 72 | | 1.85 | | | GGA(G) | 121 | | 2.53 | |
| GUG(V) | 3 | | 0.09 | | GCG(A) | | 3 | | 0.1 | | | GAG(E) | 6 | | 0.15 | | | GGG(G) | 16 | | 0.34 | |
| Average# codons=3715 | | | | | | | | | | | | | | | | | | | | | | |
|  | | | | | | | | | | | | | | | | | | | | | | |
| Codon usage of protein genes in *Narosa nigrisigna* mitogenome | | | | | | | | | | | | | | | | | | | | | | |
| Codon Table: 5 | | | | | | | | | | | | | | | | | | | | | | |
| Domain: Data | | | | | | | | | | | | | | | | | | | | | | |
| Codon | Count | | RSCU | | Codon | | Count | | RSCU | | | Codon | Count | | RSCU | | | Codon | Count | | RSCU | |
| UUU(F) | 358 | | 1.83 | | UCU(S) | | 127 | | 3.19 | | | UAU(Y) | 168 | | 1.81 | | | UGU(C) | 27 | | 1.74 | |
| UUC(F) | 33 | | 0.17 | | UCC(S) | | 5 | | 0.13 | | | UAC(Y) | 18 | | 0.19 | | | UGC(C) | 4 | | 0.26 | |
| UUA(L) | 464 | | 5.14 | | UCA(S) | | 70 | | 1.76 | | | UAA(*) | 10 | | 2 | | | UGA(W) | 94 | | 1.96 | |
| UUG(L) | 10 | | 0.11 | | UCG(S) | | 3 | | 0.08 | | | UAG(*) | 0 | | 0 | | | UGG(W) | 2 | | 0.04 | |
| CUU(L) | 47 | | 0.52 | | CCU(P) | | 84 | | 2.71 | | | CAU(H) | 62 | | 1.82 | | | CGU(R) | 18 | | 1.36 | |
| CUC(L) | 3 | | 0.03 | | CCC(P) | | 11 | | 0.35 | | | CAC(H) | 6 | | 0.18 | | | CGC(R) | 0 | | 0 | |
| CUA(L) | 18 | | 0.2 | | CCA(P) | | 27 | | 0.87 | | | CAA(Q) | 59 | | 1.93 | | | CGA(R) | 33 | | 2.49 | |
| CUG(L) | 0 | | 0 | | CCG(P) | | 2 | | 0.06 | | | CAG(Q) | 2 | | 0.07 | | | CGG(R) | 2 | | 0.15 | |
| AUU(I) | 450 | | 1.94 | | ACU(T) | | 91 | | 2.6 | | | AAU(N) | 242 | | 1.86 | | | AGU(S) | 28 | | 0.7 | |
| AUC(I) | 15 | | 0.06 | | ACC(T) | | 5 | | 0.14 | | | AAC(N) | 18 | | 0.14 | | | AGC(S) | 3 | | 0.08 | |
| AUA(M) | 252 | | 1.84 | | ACA(T) | | 44 | | 1.26 | | | AAA(K) | 103 | | 1.93 | | | AGA(S) | 82 | | 2.06 | |
| AUG(M) | 22 | | 0.16 | | ACG(T) | | 0 | | 0 | | | AAG(K) | 4 | | 0.07 | | | AGG(S) | 0 | | 0 | |
| GUU(V) | 63 | | 1.77 | | GCU(A) | | 79 | | 2.51 | | | GAU(D) | 55 | | 1.75 | | | GGU(G) | 38 | | 0.78 | |
| GUC(V) | 3 | | 0.08 | | GCC(A) | | 8 | | 0.25 | | | GAC(D) | 8 | | 0.25 | | | GGC(G) | 2 | | 0.04 | |
| GUA(V) | 68 | | 1.92 | | GCA(A) | | 37 | | 1.17 | | | GAA(E) | 64 | | 1.8 | | | GGA(G) | 126 | | 2.58 | |
| GUG(V) | 8 | | 0.23 | | GCG(A) | | 2 | | 0.06 | | | GAG(E) | 7 | | 0.2 | | | GGG(G) | 29 | | 0.59 | |
| Average# codons=3723 | | | | | | | | | | | | | | | | | | | | | | |
|  | | | | | | | | | | | | | | | | | | | | | | |
| Codon usage of protein genes in *Latoia hilarata* mitogenome | | | | | | | | | | | | | | | | | | | | | | |
| Codon Table: 5 | | | | | | | | | | | | | | | | | | | | | | |
| Domain: Data | | | | | | | | | | | | | | | | | | | | | | |
| Codon | Count | | RSCU | | Codon | | Count | | RSCU | | | Codon | Count | | RSCU | | | Codon | Count | | RSCU | |
| UUU(F) | 365 | | 1.86 | | UCU(S) | | 104 | | 2.65 | | | UAU(Y) | 156 | | 1.8 | | | UGU(C) | 29 | | 1.76 | |
| UUC(F) | 27 | | 0.14 | | UCC(S) | | 21 | | 0.54 | | | UAC(Y) | 17 | | 0.2 | | | UGC(C) | 4 | | 0.24 | |
| UUA(L) | 485 | | 5.25 | | UCA(S) | | 82 | | 2.09 | | | UAA(*) | 9 | | 2 | | | UGA(W) | 91 | | 1.94 | |
| UUG(L) | 12 | | 0.13 | | UCG(S) | | 1 | | 0.03 | | | UAG(*) | 0 | | 0 | | | UGG(W) | 3 | | 0.06 | |
| CUU(L) | 36 | | 0.39 | | CCU(P) | | 58 | | 1.86 | | | CAU(H) | 55 | | 1.57 | | | CGU(R) | 7 | | 0.54 | |
| CUC(L) | 2 | | 0.02 | | CCC(P) | | 15 | | 0.48 | | | CAC(H) | 15 | | 0.43 | | | CGC(R) | 0 | | 0 | |
| CUA(L) | 19 | | 0.21 | | CCA(P) | | 52 | | 1.66 | | | CAA(Q) | 60 | | 1.94 | | | CGA(R) | 41 | | 3.15 | |
| CUG(L) | 0 | | 0 | | CCG(P) | | 0 | | 0 | | | CAG(Q) | 2 | | 0.06 | | | CGG(R) | 4 | | 0.31 | |
| AUU(I) | 425 | | 1.87 | | ACU(T) | | 66 | | 1.93 | | | AAU(N) | 245 | | 1.85 | | | AGU(S) | 13 | | 0.33 | |
| AUC(I) | 30 | | 0.13 | | ACC(T) | | 11 | | 0.32 | | | AAC(N) | 20 | | 0.15 | | | AGC(S) | 1 | | 0.03 | |
| AUA(M) | 262 | | 1.81 | | ACA(T) | | 58 | | 1.69 | | | AAA(K) | 104 | | 1.84 | | | AGA(S) | 92 | | 2.34 | |
| AUG(M) | 27 | | 0.19 | | ACG(T) | | 2 | | 0.06 | | | AAG(K) | 9 | | 0.16 | | | AGG(S) | 0 | | 0 | |
| GUU(V) | 65 | | 1.97 | | GCU(A) | | 76 | | 2.47 | | | GAU(D) | 58 | | 1.93 | | | GGU(G) | 43 | | 0.88 | |
| GUC(V) | 2 | | 0.06 | | GCC(A) | | 2 | | 0.07 | | | GAC(D) | 2 | | 0.07 | | | GGC(G) | 4 | | 0.08 | |
| GUA(V) | 59 | | 1.79 | | GCA(A) | | 40 | | 1.3 | | | GAA(E) | 67 | | 1.79 | | | GGA(G) | 118 | | 2.41 | |
| GUG(V) | 6 | | 0.18 | | GCG(A) | | 5 | | 0.16 | | | GAG(E) | 8 | | 0.21 | | | GGG(G) | 31 | | 0.63 | |
| Average# codons=3723 | | | | | | | | | | | | | | | | | | | | | | |
|  | | | | | | | | | | | | | | | | | | | | | | |
| Codon usage of protein genes in *Iragoides fasciata* mitogenome | | | | | | | | | | | | | | | | | | | | | | |
| Codon Table: 5 | | | | | | | | | | | | | | | | | | | | | | |
| Domain: Data | | | | | | | | | | | | | | | | | | | | | | |
| Codon | Count | | RSCU | | Codon | | Count | | RSCU | | | Codon | Count | | RSCU | | | Codon | Count | | RSCU | |
| UUU(F) | 386 | | 1.88 | | UCU(S) | | 105 | | 2.58 | | | UAU(Y) | 176 | | 1.96 | | | UGU(C) | 31 | | 1.82 | |
| UUC(F) | 24 | | 0.12 | | UCC(S) | | 10 | | 0.25 | | | UAC(Y) | 4 | | 0.04 | | | UGC(C) | 3 | | 0.18 | |
| UUA(L) | 461 | | 5.17 | | UCA(S) | | 91 | | 2.23 | | | UAA(*) | 10 | | 2 | | | UGA(W) | 97 | | 2 | |
| UUG(L) | 10 | | 0.11 | | UCG(S) | | 1 | | 0.02 | | | UAG(*) | 0 | | 0 | | | UGG(W) | 0 | | 0 | |
| CUU(L) | 43 | | 0.48 | | CCU(P) | | 71 | | 2.37 | | | CAU(H) | 63 | | 1.83 | | | CGU(R) | 13 | | 1 | |
| CUC(L) | 3 | | 0.03 | | CCC(P) | | 8 | | 0.27 | | | CAC(H) | 6 | | 0.17 | | | CGC(R) | 0 | | 0 | |
| CUA(L) | 18 | | 0.2 | | CCA(P) | | 40 | | 1.33 | | | CAA(Q) | 64 | | 1.97 | | | CGA(R) | 35 | | 2.69 | |
| CUG(L) | 0 | | 0 | | CCG(P) | | 1 | | 0.03 | | | CAG(Q) | 1 | | 0.03 | | | CGG(R) | 4 | | 0.31 | |
| AUU(I) | 439 | | 1.91 | | ACU(T) | | 81 | | 2.53 | | | AAU(N) | 253 | | 1.9 | | | AGU(S) | 21 | | 0.52 | |
| AUC(I) | 20 | | 0.09 | | ACC(T) | | 6 | | 0.19 | | | AAC(N) | 14 | | 0.1 | | | AGC(S) | 2 | | 0.05 | |
| AUA(M) | 271 | | 1.88 | | ACA(T) | | 41 | | 1.28 | | | AAA(K) | 106 | | 1.93 | | | AGA(S) | 96 | | 2.36 | |
| AUG(M) | 18 | | 0.12 | | ACG(T) | | 0 | | 0 | | | AAG(K) | 4 | | 0.07 | | | AGG(S) | 0 | | 0 | |
| GUU(V) | 64 | | 1.92 | | GCU(A) | | 71 | | 2.41 | | | GAU(D) | 51 | | 1.7 | | | GGU(G) | 33 | | 0.69 | |
| GUC(V) | 2 | | 0.06 | | GCC(A) | | 9 | | 0.31 | | | GAC(D) | 9 | | 0.3 | | | GGC(G) | 1 | | 0.02 | |
| GUA(V) | 66 | | 1.98 | | GCA(A) | | 36 | | 1.22 | | | GAA(E) | 69 | | 1.86 | | | GGA(G) | 128 | | 2.67 | |
| GUG(V) | 1 | | 0.03 | | GCG(A) | | 2 | | 0.07 | | | GAG(E) | 5 | | 0.14 | | | GGG(G) | 30 | | 0.62 | |
| Average# codons=3728 | | | | | | | | | | | | | | | | | | | | | | |
|  | | | | | | | | | | | | | | | | | | | | | | |
| Codon usage of protein genes in *Quasithosea sythoffi* mitogenome | | | | | | | | | | | | | | | | | | | | | | |
| Codon Table: 5 | | | | | | | | | | | | | | | | | | | | | | |
| Domain: Data | | | | | | | | | | | | | | | | | | | | | | |
| Codon | Count | | RSCU | | Codon | | Count | | RSCU | | | Codon | Count | | RSCU | | | Codon | Count | | RSCU | |
| UUU(F) | 371 | | 1.86 | | UCU(S) | | 107 | | 2.59 | | | UAU(Y) | 168 | | 1.85 | | | UGU(C) | 34 | | 2 | |
| UUC(F) | 27 | | 0.14 | | UCC(S) | | 15 | | 0.36 | | | UAC(Y) | 14 | | 0.15 | | | UGC(C) | 0 | | 0 | |
| UUA(L) | 460 | | 5.11 | | UCA(S) | | 87 | | 2.1 | | | UAA(*) | 10 | | 2 | | | UGA(W) | 93 | | 1.96 | |
| UUG(L) | 9 | | 0.1 | | UCG(S) | | 2 | | 0.05 | | | UAG(*) | 0 | | 0 | | | UGG(W) | 2 | | 0.04 | |
| CUU(L) | 43 | | 0.48 | | CCU(P) | | 59 | | 1.9 | | | CAU(H) | 62 | | 1.8 | | | CGU(R) | 16 | | 1.21 | |
| CUC(L) | 2 | | 0.02 | | CCC(P) | | 16 | | 0.52 | | | CAC(H) | 7 | | 0.2 | | | CGC(R) | 1 | | 0.08 | |
| CUA(L) | 25 | | 0.28 | | CCA(P) | | 49 | | 1.58 | | | CAA(Q) | 64 | | 1.91 | | | CGA(R) | 34 | | 2.57 | |
| CUG(L) | 1 | | 0.01 | | CCG(P) | | 0 | | 0 | | | CAG(Q) | 3 | | 0.09 | | | CGG(R) | 2 | | 0.15 | |
| AUU(I) | 424 | | 1.87 | | ACU(T) | | 77 | | 2.3 | | | AAU(N) | 242 | | 1.86 | | | AGU(S) | 29 | | 0.7 | |
| AUC(I) | 30 | | 0.13 | | ACC(T) | | 7 | | 0.21 | | | AAC(N) | 18 | | 0.14 | | | AGC(S) | 3 | | 0.07 | |
| AUA(M) | 251 | | 1.83 | | ACA(T) | | 45 | | 1.34 | | | AAA(K) | 96 | | 1.75 | | | AGA(S) | 88 | | 2.13 | |
| AUG(M) | 24 | | 0.17 | | ACG(T) | | 5 | | 0.15 | | | AAG(K) | 14 | | 0.25 | | | AGG(S) | 0 | | 0 | |
| GUU(V) | 73 | | 2.06 | | GCU(A) | | 68 | | 2.27 | | | GAU(D) | 56 | | 1.87 | | | GGU(G) | 59 | | 1.22 | |
| GUC(V) | 2 | | 0.06 | | GCC(A) | | 10 | | 0.33 | | | GAC(D) | 4 | | 0.13 | | | GGC(G) | 2 | | 0.04 | |
| GUA(V) | 59 | | 1.66 | | GCA(A) | | 40 | | 1.33 | | | GAA(E) | 65 | | 1.71 | | | GGA(G) | 110 | | 2.27 | |
| GUG(V) | 8 | | 0.23 | | GCG(A) | | 2 | | 0.07 | | | GAG(E) | 11 | | 0.29 | | | GGG(G) | 23 | | 0.47 | |
| Average# codons=3728 | | | | | | | | | | | | | | | | | | | | | | |
|  | | | | | | | | | | | | | | | | | | | | | | |
| Codon usage of protein genes in *Monema flavescens* mitogenome | | | | | | | | | | | | | | | | | | | | | | |
| Codon Table: 5 | | | | | | | | | | | | | | | | | | | | | | |
| Domain: Data | | | | | | | | | | | | | | | | | | | | | | |
| Codon | Count | | RSCU | | Codon | | Count | | RSCU | | | Codon | Count | | RSCU | | | Codon | Count | | RSCU | |
| UUU(F) | 370 | | 1.89 | | UCU(S) | | 99 | | 2.39 | | | UAU(Y) | 153 | | 1.82 | | | UGU(C) | 29 | | 1.76 | |
| UUC(F) | 22 | | 0.11 | | UCC(S) | | 14 | | 0.34 | | | UAC(Y) | 15 | | 0.18 | | | UGC(C) | 4 | | 0.24 | |
| UUA(L) | 459 | | 5.1 | | UCA(S) | | 99 | | 2.39 | | | UAA(*) | 10 | | 2 | | | UGA(W) | 89 | | 1.87 | |
| UUG(L) | 25 | | 0.28 | | UCG(S) | | 4 | | 0.1 | | | UAG(*) | 0 | | 0 | | | UGG(W) | 6 | | 0.13 | |
| CUU(L) | 33 | | 0.37 | | CCU(P) | | 57 | | 1.81 | | | CAU(H) | 62 | | 1.77 | | | CGU(R) | 18 | | 1.38 | |
| CUC(L) | 0 | | 0 | | CCC(P) | | 13 | | 0.41 | | | CAC(H) | 8 | | 0.23 | | | CGC(R) | 2 | | 0.15 | |
| CUA(L) | 23 | | 0.26 | | CCA(P) | | 56 | | 1.78 | | | CAA(Q) | 60 | | 1.88 | | | CGA(R) | 32 | | 2.46 | |
| CUG(L) | 0 | | 0 | | CCG(P) | | 0 | | 0 | | | CAG(Q) | 4 | | 0.12 | | | CGG(R) | 0 | | 0 | |
| AUU(I) | 424 | | 1.86 | | ACU(T) | | 76 | | 2.14 | | | AAU(N) | 225 | | 1.75 | | | AGU(S) | 18 | | 0.43 | |
| AUC(I) | 31 | | 0.14 | | ACC(T) | | 11 | | 0.31 | | | AAC(N) | 32 | | 0.25 | | | AGC(S) | 1 | | 0.02 | |
| AUA(M) | 238 | | 1.76 | | ACA(T) | | 53 | | 1.49 | | | AAA(K) | 93 | | 1.69 | | | AGA(S) | 97 | | 2.34 | |
| AUG(M) | 32 | | 0.24 | | ACG(T) | | 2 | | 0.06 | | | AAG(K) | 17 | | 0.31 | | | AGG(S) | 0 | | 0 | |
| GUU(V) | 64 | | 1.74 | | GCU(A) | | 72 | | 2.44 | | | GAU(D) | 55 | | 1.83 | | | GGU(G) | 55 | | 1.11 | |
| GUC(V) | 0 | | 0 | | GCC(A) | | 9 | | 0.31 | | | GAC(D) | 5 | | 0.17 | | | GGC(G) | 4 | | 0.08 | |
| GUA(V) | 81 | | 2.2 | | GCA(A) | | 34 | | 1.15 | | | GAA(E) | 66 | | 1.71 | | | GGA(G) | 111 | | 2.23 | |
| GUG(V) | 2 | | 0.05 | | GCG(A) | | 3 | | 0.1 | | | GAG(E) | 11 | | 0.29 | | | GGG(G) | 29 | | 0.58 | |
| Average# codons=3717 | | | | | | | | | | | | | | | | | | | | | | |
|  | | | | | | | | | | | | | | | | | | | | | | |
| Codon usage of protein genes in *Parasa consocia* mitogenome | | | | | | | | | | | | | | | | | | | | | | |
| Codon Table: 5 | | | | | | | | | | | | | | | | | | | | | | |
| Domain: Data | | | | | | | | | | | | | | | | | | | | | | |
| Codon | Count | | RSCU | | Codon | | Count | | RSCU | | | Codon | Count | | RSCU | | | Codon | Count | | RSCU | |
| UUU(F) | 371 | | 1.87 | | UCU(S) | | 98 | | 2.4 | | | UAU(Y) | 151 | | 1.77 | | | UGU(C) | 31 | | 1.82 | |
| UUC(F) | 26 | | 0.13 | | UCC(S) | | 20 | | 0.49 | | | UAC(Y) | 20 | | 0.23 | | | UGC(C) | 3 | | 0.18 | |
| UUA(L) | 459 | | 4.98 | | UCA(S) | | 88 | | 2.15 | | | UAA(*) | 9 | | 2 | | | UGA(W) | 90 | | 1.94 | |
| UUG(L) | 25 | | 0.27 | | UCG(S) | | 4 | | 0.1 | | | UAG(*) | 0 | | 0 | | | UGG(W) | 3 | | 0.06 | |
| CUU(L) | 38 | | 0.41 | | CCU(P) | | 60 | | 1.94 | | | CAU(H) | 63 | | 1.8 | | | CGU(R) | 7 | | 0.54 | |
| CUC(L) | 9 | | 0.1 | | CCC(P) | | 20 | | 0.65 | | | CAC(H) | 7 | | 0.2 | | | CGC(R) | 2 | | 0.15 | |
| CUA(L) | 22 | | 0.24 | | CCA(P) | | 42 | | 1.35 | | | CAA(Q) | 56 | | 1.84 | | | CGA(R) | 41 | | 3.15 | |
| CUG(L) | 0 | | 0 | | CCG(P) | | 2 | | 0.06 | | | CAG(Q) | 5 | | 0.16 | | | CGG(R) | 2 | | 0.15 | |
| AUU(I) | 411 | | 1.87 | | ACU(T) | | 74 | | 2.07 | | | AAU(N) | 229 | | 1.84 | | | AGU(S) | 14 | | 0.34 | |
| AUC(I) | 29 | | 0.13 | | ACC(T) | | 10 | | 0.28 | | | AAC(N) | 20 | | 0.16 | | | AGC(S) | 1 | | 0.02 | |
| AUA(M) | 267 | | 1.84 | | ACA(T) | | 56 | | 1.57 | | | AAA(K) | 106 | | 1.83 | | | AGA(S) | 101 | | 2.47 | |
| AUG(M) | 23 | | 0.16 | | ACG(T) | | 3 | | 0.08 | | | AAG(K) | 10 | | 0.17 | | | AGG(S) | 1 | | 0.02 | |
| GUU(V) | 68 | | 2.06 | | GCU(A) | | 71 | | 2.35 | | | GAU(D) | 54 | | 1.83 | | | GGU(G) | 43 | | 0.88 | |
| GUC(V) | 1 | | 0.03 | | GCC(A) | | 5 | | 0.17 | | | GAC(D) | 5 | | 0.17 | | | GGC(G) | 3 | | 0.06 | |
| GUA(V) | 62 | | 1.88 | | GCA(A) | | 40 | | 1.32 | | | GAA(E) | 60 | | 1.62 | | | GGA(G) | 118 | | 2.42 | |
| GUG(V) | 1 | | 0.03 | | GCG(A) | | 5 | | 0.17 | | | GAG(E) | 14 | | 0.38 | | | GGG(G) | 31 | | 0.64 | |
| Average# codons=3710 | | | | | | | | | | | | | | | | | | | | | | |
|  | | | | | | | | | | | | | | | | | | | | | | |
| Codon usage of protein genes in *Apoda limacodes* mitogenome | | | | | | | | | | | | | | | | | | | | | | |
| Codon Table: 5 | | | | | | | | | | | | | | | | | | | | | | |
| Domain: Data | | | | | | | | | | | | | | | | | | | | | | |
| Codon | Count | | RSCU | | Codon | | Count | | RSCU | | | Codon | Count | | RSCU | | | Codon | Count | | RSCU | |
| UUU(F) | 370 | | 1.89 | | UCU(S) | | 125 | | 3.06 | | | UAU(Y) | 161 | | 1.75 | | | UGU(C) | 30 | | 1.88 | |
| UUC(F) | 21 | | 0.11 | | UCC(S) | | 10 | | 0.24 | | | UAC(Y) | 23 | | 0.25 | | | UGC(C) | 2 | | 0.13 | |
| UUA(L) | 482 | | 5.23 | | UCA(S) | | 79 | | 1.93 | | | UAA(*) | 9 | | 2 | | | UGA(W) | 93 | | 1.94 | |
| UUG(L) | 7 | | 0.08 | | UCG(S) | | 1 | | 0.02 | | | UAG(*) | 0 | | 0 | | | UGG(W) | 3 | | 0.06 | |
| CUU(L) | 44 | | 0.48 | | CCU(P) | | 77 | | 2.5 | | | CAU(H) | 66 | | 1.86 | | | CGU(R) | 17 | | 1.31 | |
| CUC(L) | 1 | | 0.01 | | CCC(P) | | 12 | | 0.39 | | | CAC(H) | 5 | | 0.14 | | | CGC(R) | 1 | | 0.08 | |
| CUA(L) | 19 | | 0.21 | | CCA(P) | | 34 | | 1.11 | | | CAA(Q) | 62 | | 1.94 | | | CGA(R) | 32 | | 2.46 | |
| CUG(L) | 0 | | 0 | | CCG(P) | | 0 | | 0 | | | CAG(Q) | 2 | | 0.06 | | | CGG(R) | 2 | | 0.15 | |
| AUU(I) | 436 | | 1.91 | | ACU(T) | | 86 | | 2.42 | | | AAU(N) | 232 | | 1.81 | | | AGU(S) | 34 | | 0.83 | |
| AUC(I) | 21 | | 0.09 | | ACC(T) | | 7 | | 0.2 | | | AAC(N) | 25 | | 0.19 | | | AGC(S) | 1 | | 0.02 | |
| AUA(M) | 249 | | 1.84 | | ACA(T) | | 48 | | 1.35 | | | AAA(K) | 100 | | 1.8 | | | AGA(S) | 77 | | 1.88 | |
| AUG(M) | 21 | | 0.16 | | ACG(T) | | 1 | | 0.03 | | | AAG(K) | 11 | | 0.2 | | | AGG(S) | 0 | | 0 | |
| GUU(V) | 70 | | 2.12 | | GCU(A) | | 81 | | 2.66 | | | GAU(D) | 56 | | 1.87 | | | GGU(G) | 53 | | 1.04 | |
| GUC(V) | 0 | | 0 | | GCC(A) | | 6 | | 0.2 | | | GAC(D) | 4 | | 0.13 | | | GGC(G) | 2 | | 0.04 | |
| GUA(V) | 62 | | 1.88 | | GCA(A) | | 33 | | 1.08 | | | GAA(E) | 68 | | 1.81 | | | GGA(G) | 136 | | 2.67 | |
| GUG(V) | 0 | | 0 | | GCG(A) | | 2 | | 0.07 | | | GAG(E) | 7 | | 0.19 | | | GGG(G) | 13 | | 0.25 | |
| Average# codons=3732 | | | | | | | | | | | | | | | | | | | | | | |

* The sequences for species with names in bold were generated in this study.
